# SSHscreen and SSHdb, generic software for microarray based gene discovery: application to the stress response in cowpea

**DOI:** 10.1186/1746-4811-6-10

**Published:** 2010-04-01

**Authors:** Nanette Coetzer, Inge Gazendam, Dean Oelofse, Dave K Berger

**Affiliations:** 1 ACGT Computational Biology and Bioinformatics Unit, Department of Biochemistry, University of Pretoria, 0002, South Africa; 2 Germplasm Development Division, Agricultural Research Council-Vegetable and Ornamental Plant Institute, Private Bag X293, Pretoria, 0001, South Africa; 3 Department of Plant Science, Forestry and Agricultural Biotechnology Institute (FABI), University of Pretoria, 0002, South Africa

## Abstract

**Background:**

Suppression subtractive hybridization is a popular technique for gene discovery from non-model organisms without an annotated genome sequence, such as cowpea (*Vigna unguiculata *(L.) Walp). We aimed to use this method to enrich for genes expressed during drought stress in a drought tolerant cowpea line. However, current methods were inefficient in screening libraries and management of the sequence data, and thus there was a need to develop software tools to facilitate the process.

**Results:**

Forward and reverse cDNA libraries enriched for cowpea drought response genes were screened on microarrays, and the R software package SSHscreen 2.0.1 was developed (i) to normalize the data effectively using spike-in control spot normalization, and (ii) to select clones for sequencing based on the calculation of enrichment ratios with associated statistics. Enrichment ratio 3 values for each clone showed that 62% of the forward library and 34% of the reverse library clones were significantly differentially expressed by drought stress (adjusted p value < 0.05). Enrichment ratio 2 calculations showed that > 88% of the clones in both libraries were derived from rare transcripts in the original tester samples, thus supporting the notion that suppression subtractive hybridization enriches for rare transcripts. A set of 118 clones were chosen for sequencing, and drought-induced cowpea genes were identified, the most interesting encoding a late embryogenesis abundant Lea5 protein, a glutathione S-transferase, a thaumatin, a universal stress protein, and a wound induced protein. A lipid transfer protein and several components of photosynthesis were down-regulated by the drought stress. Reverse transcriptase quantitative PCR confirmed the enrichment ratio values for the selected cowpea genes. SSHdb, a web-accessible database, was developed to manage the clone sequences and combine the SSHscreen data with sequence annotations derived from BLAST and Blast2GO. The self-BLAST function within SSHdb grouped redundant clones together and illustrated that the SSHscreen plots are a useful tool for choosing anonymous clones for sequencing, since redundant clones cluster together on the enrichment ratio plots.

**Conclusions:**

We developed the SSHscreen-SSHdb software pipeline, which greatly facilitates gene discovery using suppression subtractive hybridization by improving the selection of clones for sequencing after screening the library on a small number of microarrays. Annotation of the sequence information and collaboration was further enhanced through a web-based SSHdb database, and we illustrated this through identification of drought responsive genes from cowpea, which can now be investigated in gene function studies. SSH is a popular and powerful gene discovery tool, and therefore this pipeline will have application for gene discovery in any biological system, particularly non-model organisms. SSHscreen 2.0.1 and a link to SSHdb are available from http://microarray.up.ac.za/SSHscreen.

## Background

A range of techniques are available for gene discovery. Expressed sequence tag (EST) sequencing of cloned cDNAs is a common approach with the advantage that if full-length cDNAs are cloned they can be directly employed for further gene function experiments [1]. Cloned cDNAs can be arrayed on high-density microarrays and used for expression profiling [2]. Next generation sequencing, such as 454 technology™, has been employed for sequencing cDNA libraries [3], and the term RNA-Seq has been dubbed for this approach when applied at deep enough coverage to compare transcript counts between one or more biological states [4]. Previous methods, such as serial analysis of gene expression (SAGE), are also based on counting short sequence tags [5]. Although these methods provided exceptional quantitative analysis, they are labour-intensive and currently very costly. Additionally, they are most effective if an annotated genome sequence is available.

Many research laboratories that are investigating non-model crops without genome sequence resources or have research questions that do not require a full genome analysis have the option of applying different "RNA fingerprinting" techniques for gene discovery. Examples of these techniques are differential display RT-PCR (DD-RT-PCR), RNA-fingerprinting by arbitrarily primed PCR (RAP-PCR) and cDNA amplified fragment length polymorphism (cDNA-AFLP) where cDNA sub populations are amplified and visualized on polyacrylamide gels, whereafter differentially expressed transcripts are isolated from the gel for sequencing [6-8]. These methods have limitations such as bias based on choice of initial primer sets, problems with reproducibility, generation of false positives, and reliance on time-consuming polyacrylamide gel electrophoresis and gel extraction to obtain sequence information. Another limitation of the above methods is the difficulty to capture low abundance clones.

A third alternative for gene discovery are PCR-based cDNA subtractive hybridization methods. These methods exclude common cDNA sequences between the two or more samples and, thus enrich for target sequences of interest, which are subsequently cloned. These methods include representational difference analysis (RDA) and suppression subtractive hybridization (SSH) [9-11]. SSH is considered an effective method to enrich for rare transcripts [10]. A recent search with the keywords 'suppression subtractive hybridization' in the title of research articles in PubMed produced 1213 hits (data not shown), indicating that SSH remains a popular method for the construction of enriched cDNA libraries. We chose to apply SSH to gene discovery in the non-model crop cowpea, and in this work we describe two software innovations that facilitate gene discovery using SSH.

Subsequent to gene cloning methods such as SSH, integrated bioinformatics tools for sequence management and annotation are needed. Various automated in-house pipelines have been developed to process and annotate EST/cDNA sequences exploiting public software, and collecting data in customized databases according to specific needs [12-14]. The cDNA Annotation System (CAS) is a useful tool for large-scale annotation, which can be implemented on a single desktop. Automatic annotations of sequences can subsequently be manually investigated and curated [15]. SSHSuite is an example of a workstation package capable of handling and storing cDNA sequences from a SSH library [16].

In this study we chose to apply SSH to gene discovery from cowpea (*Vigna unguiculata *(L.) Walp.) plants. Cowpea is a tropical legume crop with a high protein content, since it is able to fix nitrogen, and is used as a protein substitute for meat products [17]. The crop is fully utilised by people in Africa as leaves and seeds are consumed, and the plants are used for grazing and the feeding of livestock. Since many lines are drought tolerant, cowpea can be grown under the harshest growing conditions, and in the poorest soils, and is, therefore, an important crop for subsistence and small-holder farmers [18]. Breeding efforts to improve yield of cowpea under different production systems is ongoing [17], and lines with differential drought tolerance have been identified [19-21]. Promising QTLs for drought tolerance in cowpea have recently been reported [22].

Cowpea can be classified as an orphan crop, which means that it is important for food security in many developing countries, however limited research funding has been devoted to it [23]. Genomics resources for cowpea are starting to be developed with sequencing of a methyl-filtered genomic library [24], as well as an EST dataset [23]. The availability of a cowpea breeding line that exhibited drought tolerance in the field prompted us to investigate gene expression in this line in response to drought stress. Based on previous experience of using SSH for gene discovery in other orphan crops, banana and pearl millet [25,26], we encountered bottlenecks in the process. Consequently, in this study we developed improvements to the gene discovery pipeline, through the software SSHscreen 2.0.1, an R package, which quantitatively describes each clone in the library in terms of up/down regulation and rarity/abundance in the treated sample. We then validated the enrichment ratio calculations from the microarray screening and SSHscreen 2.0.1 analysis for selected drought-responsive cowpea clones using quantitative PCR (qPCR). SSHscreen facilitated the efficient choice of clones to be sequenced, which then led to the development of a web-based sequence database SSHdb, which facilitated the management and annotation of the SSH cDNA library clones. We, therefore, report development of the SSHscreen-SSHdb pipeline, a useful resource for any research group embarking on gene discovery using SSH.

## Results

### Construction of cowpea drought expression SSH library and overview of SSHscreen/SSHdb data analysis pipeline

**Figure 1:**
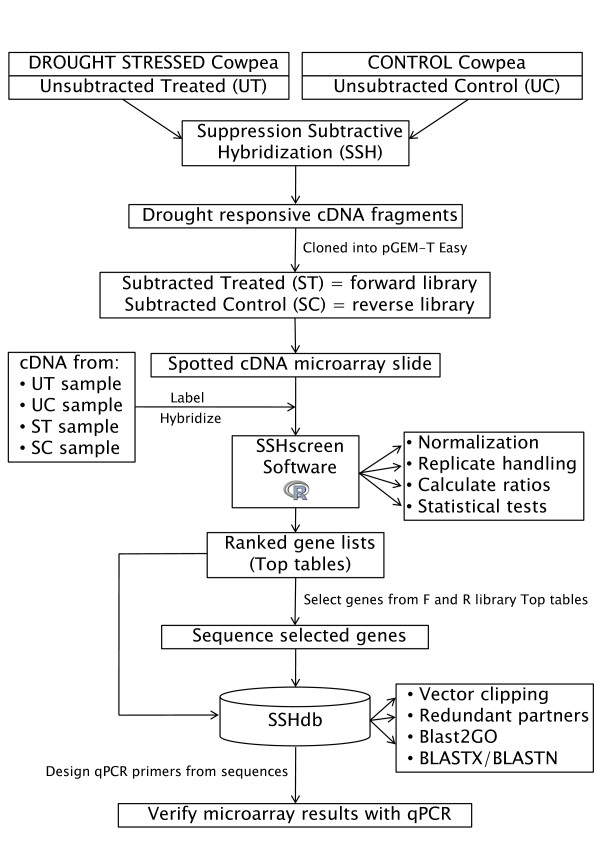
**Schematic representation of the flow of data through the SSHscreen-SSHdb pipeline**. SSH was used for the construction of a cowpea drought expression library. Tester cDNA were prepared from drought stressed IT96D-602 cowpea leaf RNA and driver cDNA from control Tvu7778 cowpea leaf RNA, and vice versa for the reverse library. The subtracted library was spotted onto glass slides and hybridised with a mix of differently labelled subtracted and unsubtracted cDNA from the two cowpea cultivars. The R package SSHscreen 2.0.1 was used to analyse the microarray data, using limma functions for pre-processing the data as well as to identify statistically significant differentially expressed genes. A subset of clones was selected for sequencing. Available FASTA sequences as well as top tables (output from SSHscreen) were uploaded to the web-based database, SSHdb, to manage and annotate clones in the library. Expression results for selected genes were verified using qPCR.

We developed a pipeline for quantitative screening and sequence management of clones from a SSH cDNA library. The pipeline is particularly useful for gene discovery in non-sequenced organisms. As an example, we used a cowpea (*V. unguiculata*, (L.) Walp) drought expression library where the objective was to identify and isolate genes responding to drought stress in cowpea. Figure 1 gives an outline of the pipeline. SSH [10] was used to enrich for genes that were differentially expressed between drought stressed and unstressed cowpea plants. Cowpea breeding lines from the International Institute of Tropical Agriculture (IITA) that were previously shown to be drought tolerant (line IT96D-602) and drought susceptible (line Tvu7778) were used [27]. The "tester" for forward library construction was from drought stressed line IT96D-602 (cDNA pooled from plants 9 and 12 days after water was withheld), whereas the "driver" for forward library construction was from control treated line Tvu7778 (cDNA pooled from plants grown for the same time on a normal watering regime). These time points were chosen for maximum drought stress symptoms, before leaves were too senesced for RNA extractions. The aim of this wide subtraction was to be sure to capture sufficient differentially expressed transcripts to illustrate the efficacy of the SSHscreen/SSHdb software. This could include not only genes that are induced/repressed by drought stress in drought tolerant IT96D-602 only, but also those that are constitutively expressed at higher/lower levels in IT96D-602 compared to the drought sensitive line Tvu7778. Good quality forward and reverse subtracted cDNA fragments were generated (data not shown) and used to construct a cDNA library with a total of 4160 cDNA clones (2144 in the forward and 2016 in the reverse library), which were amplified by PCR and spotted in duplicate onto glass slides for screening and selection of clones for sequencing [28](Figure 1).

Subtracted and unsubtracted cDNA samples from cowpea used to construct the SSH libraries were prepared as Cy3- and Cy5-labelled targets and hybridized to the microarrays. These cDNA samples were UT (unsubtracted treated), UC (unsubtracted control), ST (subtracted treated, i.e. forward library), and SC (subtracted control, i.e. reverse library)(Figure 1). The R package SSHscreen version 2.0.1, available from http://microarray.up.ac.za/SSHscreen/, was developed in this study to analyse the resulting microarray data using limma functions, thereby quantitatively screening the library for significantly differentially expressed clones [28](Figure 1). SSHscreen analysis of the microarray data was used to assist in selection of 118 clones for sequencing, based on their statistics of differential expression (Figure 1). The SSHscreen data output (top tables with the statistics of differential expression for each clone), as well as the selected sequences in FASTA format were uploaded to SSHdb. SSHdb was developed as a web-based database for sequence management and annotation of clones in SSH libraries and can be accessed at http://sshdb.bi.up.ac.za/ (Figure 1). A screenshot of the SSHdb interface is given in Additional file 1, showing data for some of the clones in the cowpea SSH library. BLAST analysis that was carried out when sequences were uploaded to SSHdb was used to combine clones with the same sequence into redundant partner groups, and Blast2GO was used to identify putative annotations for each group. Six genes identified from the cowpea SSH library were selected and used to validate the microarray/SSHscreen results with an independent technique - qPCR (Figure 1).

### Screening the cowpea SSH libraries using SSHscreen 2.0.1

SSHscreen facilitates the screening of an SSH library using cDNA microarrays [28]. Each clone was quantitatively described in terms of up/down regulation (Enrichment ratio 3 (ER3) values: log_2_(UT/UC) for forward library; log_2_(UC/UT) for reverse library) and rarity/abundance (Enrichment ratio 2 (inverse ER2) values: log_2_(UT/ST) for forward library; log_2_(UC/SC) for reverse library) in the treated sample; and a measure of statistical significance for each result was provided in the form of a moderated t-statistic with an associated p-value [29]. SSHscreen was built around the limma R package from the BioConductor project [29], which provides the functionality for importing and analyzing gene expression microarray data. Berger *et al*. [28] described the implementation of the original version of SSHscreen (version 1.0.1). An improved version, SSHscreen 2.0.1 was developed to analyse the data from the cowpea SSH libraries in this study.

High quality microarray images were obtained from hybridization of pairs of Cy-labelled cDNA targets (UT, UC, ST or SC) to the cowpea drought expression microarrays. The average numbers of spots across the 12 arrays with signal-to-noise ratios > 3 were 83% and 85% for the Cy3 and Cy5 channels, respectively. The average coefficients of variance (%CV)(standard deviation*100/mean intensity) for the background values across the 12 arrays were 7 and 12% for the Cy3 and Cy5 channels, respectively. This is supported by visual inspection of pseudocolour images of slides. For example, strong hybridization of Cy3 targets from ST to probes from the forward library spotted in the top six rows of each array block can be observed as green spots in Additional file 2a, whereas Cy5 targets from UT hybridize predominantly to probes from the reverse library as red spots (rows 7-11 of each array block) (Additional file 2a). The opposite hybridization pattern is observed in a dye swap slide (Additional file 2b), as expected.

Within-slide normalization of two-colour microarray data is an important consideration to account for systematic bias due to differences between the Cy3 and Cy5 dyes [30]. Commonly, loess normalization is applied [30], however this is based on the assumption that most of the genes on the array are not differentially expressed. This is legitimate for most whole genome microarray experiments, however it is not appropriate when the array is constructed from an SSH library, which selects for differentially expressed genes. Therefore, spike-in control spot-based normalization was applied in SSHscreen analysis of the cowpea SSH libraries [30]. Serial dilutions of four "alien" control probes (green fluorescent protein (*gfp*), human beta-globin (*globin*), bacterial neomycin phosphotransferase II (*nptII) *and a fungal rRNA gene internal transcribed spacer (*its*); see methods) were spotted on the glass slides. These probes were chosen since matching sequences were unlikely to be present in the cowpea cDNA samples. Importantly, a "spike-in" control mix of fragments of the genes corresponding to the four control probes *gfp, globin, nptII *and *its *was prepared in which each of the four genes was present at a different concentration. The spike-in control mix was added in equal amounts to each cDNA target sample prior to labelling. The dilution series of control spots on each array which have hybridized to the spike-in controls (added in equal amounts to the pairs of target cDNAs) can be observed in the raw pseudocolour images as yellow spots in row 12 of most array blocks (Additional file 2).

Within-slide normalization using the spike-in control spots was effective in our study, and this was illustrated by boxplots of the control spots across the 12 slides, which showed that the variability of M values in the raw data was diminished considerably by the normalization (Additional file 3). The average standard deviations of the M values for the control spots across the 12 slides decreased from 0.18 to 0.10 after normalization, a similar improvement to that reported in Figure 2 of Fardin et al. [31], who also applied control spot-based normalization. This can also be visualized in the MA plots (Additional file 4), since the control spots (colours other than blue or yellow) were placed on M = 0 line in the MA-plots after normalization. Clones of the forward and reverse libraries were illustrated by blue and yellow dots in Additional file 4 panels a-h and i-p, respectively. Dye swap slides showed consistent clouds of data points above and below the M = 0 line, as expected (compare panel a with b, for example). Effective normalization would also be expected to decrease the variation of the M values for the clones. The average standard deviations of individual clone M values decreased by 37% and 8% for the ER3 and ER2 slides, respectively. We also calculated the consistency of clone spot intensities across pairs of replicate slides, and Pearson's correlation coefficients ranged from 0.78 (Additional file 4i vs. 4k) to 0.96 (Additional file 4f vs 4h) with an average of 0.85 (data not shown).

**Figure 2:**
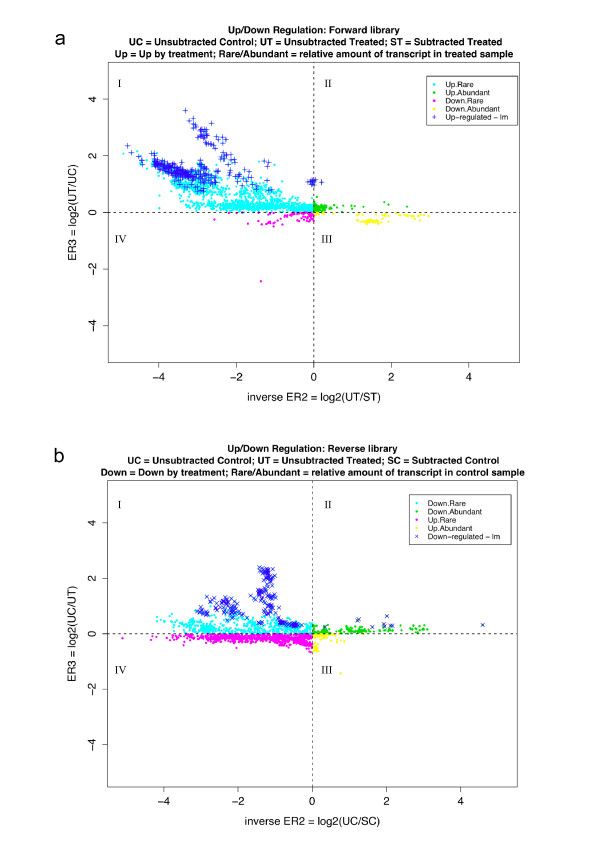
**ER3 versus inverse ER2 plot produced by SSHscreen for the cowpea forward (a) and reverse (b) libraries**. **(a) **ER3 for the forward library was calculated as the log-2 ratio of the unsubtracted treated cDNA (UT; drought stressed sample) divided by the unsubtracted control cDNA (UC). Inverse ER2 was calculated as the log-2 ratio of UT divided by the subtracted treated cDNA (ST; SSH library enriched for genes up-regulated by drought stress). Data points were classified as representing transcripts: up-regulated by stress treatment/rare (Up.Rare: quadrant 1; ER3 > 0 and inverse ER2 < 0); up-regulated by stress treatment/abundant (Up.Abundant: quadrant 2; ER3 > 0 and inverse ER2 > 0); down-regulated by stress treatment/rare (Down.Rare: quadrant 3; ER3 < 0 and inverse ER2 > 0); and down-regulated by stress treatment/abundant (Down.Abundant: quadrant 4; ER3 < 0 and inverse ER2 < 0). The top 300 statistically significant clones are represented on the plot (adjusted p value < 0.05). **(b) **ER3 for the reverse library was calculated as the log-2 ratio of the unsubtracted control cDNA (UC) divided by the unsubtracted treated cDNA (UT; drought stressed). Inverse ER2 was calculated as the log-2 ratio of the unsubtracted control cDNA (UC) divided by the subtracted control cDNA (SC; SSH library enriched for genes down-regulated by drought stress). Data points were classified as representing transcripts: down-regulated by stress treatment/rare (Down.Rare: quadrant 1; ER3 > 0 and inverse ER2 < 0); down-regulated by stress treatment/abundant (Down.Abundant: quadrant 2; ER3 > 0 and inverse ER2 > 0); up-regulated by stress treatment/rare (Up.Rare: quadrant 3; ER3 < 0 and inverse ER2 > 0); and up-regulated by stress treatment/abundant (Up.Abundant: quadrant 4; ER3 < 0 and inverse ER2 < 0). The top 300 statistically significant clones are represented on the plot (adjusted p value < 0.05).

The results of the SSHscreen 2.0.1 analysis were visualised by ER3 versus inverse ER2 plots for the forward and reverse libraries (Figure 2a and Figure 2b, respectively). Most of the genes in these plots fall in quadrant I, where ER3 > 0 and inverse ER2 < 0, meaning up-regulated by drought stress and rare in the unsubtracted drought stressed cDNA for the forward library (92%; Figure 2a), and down-regulated by drought stress and rare in the control cDNA for the reverse library (52%; Figure 2b). The criterion we chose to score genes as statistically significant differentially expressed (ER3 analysis: UT versus UC comparison) was that the adjusted p-value should be less than 0.05 after the linear model fit and empirical Bayes calculations. The p-value reflects the probability of rejecting the null hypothesis that there is no differential expression between the drought stressed (UT) and control (UC) samples for the forward library, and vice versa for the reverse library. There were 62% (1337/2146) significantly differentially expressed clones in the forward library and 34% (688/2018) in the reverse library using the stringent criterion of adjusted p value < 0.05. Only the most significant 300 for each library are marked in Figure 2a and 2b. The quality of the subtraction process in construction of the forward library was reflected in the low number of clones that had negative ER3 values (8%; Figure 2a), whereas the subtraction was less efficient for the reverse library (48% with negative ER3 values; Figure 2b).

SSHscreen also provides an alternative statistic to choose differentially expressed genes, namely the B-statistic, by implementing this function of limma. The B-statistic [32,33] can be interpreted as the log-odds that a specific gene is differentially expressed. This means that a B-statistic of zero corresponds to a 50-50 chance of differential expression, and accordingly a user is generally interested in genes with a positive B-statistic. For the cowpea ER3 analysis, 67% of the clones in the forward and 52% of the clones in the reverse library had positive B-statistics, which are larger numbers of clones than those selected based on the stringent criterion of t-statistic adjusted pvalue < 0.05.

Table 1 shows the top 20 cowpea clones sorted by p-value for the forward and reverse libraries extracted from the top tables that were generated from the ER3 analysis in SSHscreen. The most significant up-regulated forward library clone, 46D03-F, had a log-2 fold change of 2.9 (equivalent to the ER3 value). Taking the antilog of the log with base 2, it can be shown that this clone was ~8-fold up-regulated. With a similar calculation it can be shown that the most significant down-regulated reverse library clone, 45C07-R, with a log-2 fold change of 2.4, was ~5-fold down-regulated. The top table also reported the Average expression (A value) and statistics associated with the ER3 value, namely a moderated t-statistic, a p-value, an adjusted p-value and a B-statistic (Table 1). A top table for the ER2 analysis was also generated by SSHscreen, which reported the statistics of whether the clones represent rare or abundant transcripts in the original treated sample (data not shown).

**Table 1:** Top tables produced by SSHscreen for the forward and reverse cowpea libraries

Forward library top table: up/down regulation							
**ID**	logFC(ER3)*	**AveExpr**	**t**	**P.Value**	**adj.P.Val**	**B**	**invER2**

46D03-F	2.91	10.89	34.37	5.4E-12	1.1E-08	19.51	-2.77
25B07-F	3.09	11.13	31.07	1.5E-11	1.6E-08	19.04	-3.06
13C10-F	2.71	10.62	29.27	2.8E-11	1.9E-08	18.73	-2.75
07E11-F	2.86	10.18	26.11	9.0E-11	3.2E-08	18.10	-2.84
13B02-F	2.77	10.59	25.71	1.1E-10	3.2E-08	18.01	-2.76
07E08-F	2.67	8.97	25.70	1.1E-10	3.2E-08	18.00	-2.51
26F04-F	2.98	11.46	25.60	1.1E-10	3.2E-08	17.98	-3.11
25A05-F	2.94	10.82	25.26	1.3E-10	3.2E-08	17.90	-2.81
25B08-F	2.86	11.25	24.99	1.4E-10	3.2E-08	17.83	-2.80
13C01-F	2.73	10.41	24.74	1.6E-10	3.2E-08	17.77	-2.77
05E09-F	2.58	10.86	23.98	2.1E-10	4.0E-08	17.58	-2.27
25C06-F	2.92	11.08	23.75	2.4E-10	4.0E-08	17.52	-2.92
25B06-F	3.60	11.17	22.99	3.3E-10	5.2E-08	17.31	-3.31
13F02-F	2.61	10.99	22.68	3.8E-10	5.2E-08	17.22	-2.84
13A08-F	2.65	10.84	22.54	4.0E-10	5.2E-08	17.18	-2.82
13C12-F	2.93	11.03	22.53	4.0E-10	5.2E-08	17.17	-2.89
33A07-F	3.32	10.42	22.27	4.5E-10	5.3E-08	17.10	-3.11
33C06-F	3.23	10.61	22.21	4.7E-10	5.3E-08	17.08	-3.22
06H12-F	2.41	10.54	21.83	5.5E-10	5.8E-08	16.96	-2.65
08B07-F	1.88	10.01	21.76	5.7E-10	5.8E-08	16.94	-2.40

**Reverse library top table: up/down regulation**							

**ID**	**logFC(ER3)**#	**AveExpr**	**t**	**P.Value**	**adj.P.Val**	**B**	**invER2**

45C07-R	2.36	11.01	26.14	2.9E-10	5.5E-07	13.66	-1.35
36E04-R	2.25	10.81	22.19	1.4E-09	7.4E-07	13.20	-1.38
36B11-R	2.33	11.31	21.49	1.9E-09	7.4E-07	13.10	-1.22
44C07-R	2.39	11.69	20.93	2.4E-09	7.4E-07	13.02	-1.42
37B05-R	2.33	10.99	20.55	2.8E-09	7.4E-07	12.96	-1.07
45B02-R	2.26	11.05	20.32	3.2E-09	7.4E-07	12.92	-1.20
35F03-R	2.28	11.24	20.11	3.5E-09	7.4E-07	12.88	-1.41
35H05-R	2.35	11.22	20.08	3.5E-09	7.4E-07	12.88	-1.23
35E05-R	2.10	10.79	19.99	3.7E-09	7.4E-07	12.86	-1.02
45E02-R	2.29	10.88	19.88	3.9E-09	7.4E-07	12.84	-1.19
45G05-R	2.18	10.27	19.26	5.2E-09	9.0E-07	12.73	-1.24
35H04-R	2.24	11.22	18.85	6.4E-09	1.0E-06	12.65	-1.29
36A03-R	2.02	10.15	18.21	8.8E-09	1.3E-06	12.51	-1.22
16D06-R	1.65	11.02	17.88	1.1E-08	1.4E-06	12.44	-1.28
16C10-R	1.98	11.01	16.74	1.9E-08	2.3E-06	12.17	-1.20
35A01-R	2.19	10.92	16.73	2.0E-08	2.3E-06	12.16	-1.27
23G10-R	1.74	10.59	16.68	2.0E-08	2.3E-06	12.15	-1.08
45C04-R	2.17	10.50	16.11	2.8E-08	2.9E-06	12.00	-1.34
37C11-R	1.99	11.20	16.06	2.9E-08	2.9E-06	11.98	-1.08
16D05-R	2.09	11.12	15.85	3.2E-08	3.1E-06	11.92	-1.01

Only the top 20 statistically significant up- and down-regulated genes (before sequencing) are shown (sorted by B statistic).

*ER3 for Forward library calculated as log_2_(UT/UC)

^#^ER3 for Reverse library calculated as log_2_(UC/UT)

### Annotation and management of cowpea SSH library sequences using SSHdb

The top tables (e.g. Table 1) and plots (Figure 2a and 2b) from SSHscreen analysis of the forward and reverse libraries were used to effectively select clones for sequencing based on the criteria of most significant differential expression and least likelihood of sequencing the same gene fragment twice. This was achieved by choosing those clones with the lowest adjusted p-value calculated from the ER3 values. Selection of clones that were spatially separated on the SSHscreen ER plots (Figure 2a and 2b) increased the likelihood of sequencing non-redundant clones. Sequence data for 118 clones, as well as SSHscreen top table data for the entire array, were uploaded to SSHdb for interpretation and management of the data.

**Figure 3:**
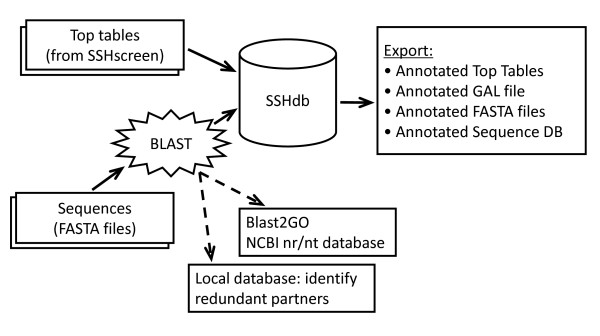
**Schematic representation of SSHdb**. Top tables from SSHscreen, as well as available FASTA sequences for individual clones can be uploaded to SSHdb. For each input FASTA sequence, BLAST searches against the local database are performed in order to identify redundant partners in the library. For each redundant partner group, a representative clone is selected and BLAST searches against the NCBI non-redundant (nr) and nucleotide (nt) databases are performed in order to annotate each group with putative functions. Output from SSHdb includes annotated top tables, annotated GAL files and annotated FASTA files. The user can also export a tab-delimited file of the annotated sequence database, containing all available information about each sequenced clone in the library.

Figure 3 gives a schematic representation of the flow of data through SSHdb. For each input cowpea sequence (in FASTA format), SSHdb removed the vector and adaptor fragments by performing BLASTN searches against the NCBI UniVec database http://www.ncbi.nlm.nih.gov/VecScreen/UniVec.html. Next, similarity searches were carried out against all sequences already uploaded in the database, in order to identify clones with the same sequence i.e. redundant partners in the library, using a BLASTN E-value cut-off of 10e-10. Thirty nine of the 118 sequenced clones were unique, implying that 67% of these sequences were redundant partners (Additional file 5). The largest group had 19 redundant partners. For each of the 39 redundant partner groups, the longest sequence in the group was selected by default as the representative clone. The choice of representative clone could be reviewed by downloading from SSHdb the multiple sequence alignments of redundant partner groups with two or more members (generated by ClustalW).

**Figure 4:**
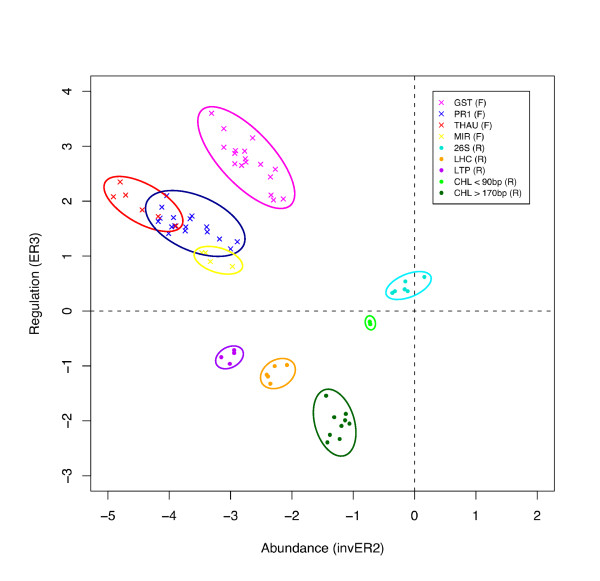
**ER3 versus inverse ER2 plot for sequenced clones to illustrate that redundant partners cluster together**. The ER-values of clones from the eight largest redundant partner groups in the library were plotted. Clones from the same redundant partner groups clustered together. Groups are colour coded and labelled as follows: glutathione S-transferase GST (mauve), pathogenesis related protein 1a (PR1; blue), Thaumatin (THAU; red), miraculin (MIR; yellow), 26S rRNA (26S; blue), light harvesting complex PSII) (LHC; orange), lipid transfer protein (LTP; purple), chlorophyll a/b-binding protein (CHL < 90 bp; green) (CHL > 170 bp; dark green).

Following the identification of redundant partner groups, annotation was performed on the representative clones, using Blast2GO [34], thereby inferring putative functions for each group (Additional file 5). BLASTN and BLASTX [35] against the NCBI non-redundant nucleotide database (nt) and the NCBI non-redundant peptide database (nr) was also carried out. For cases where the E-value of the top BLASTX hit was low enough (less than 10e-10), this hit was automatically selected as the default priority annotation. Blast2GO and the top 10 BLASTX and BLASTN hits were stored in the database. For each redundant partner group, SSHdb allowed the top BLAST results to be viewed and in several cases the priority annotation was changed after manual inspection. SSHdb linked the selected BLAST annotations to SSHscreen top table entries and it was possible to export different combinations of annotation information for selected subsets of clones (for example, this allowed the construction of Figure 4, see later). One could export selected clones as FASTA files with the functional annotation as part of the header, which was particularly useful in preparing the sequences for submission to GenBank, or as a tab delimited text file containing various columns of available annotation information linked to the selected clones (Additional file 5). SSHdb also provides the option to export annotated SSHscreen top tables or Genepix Array List (GAL) files.

### Cowpea SSH library contains genes known to play a role in plant response to stress

Additional file 5 is a summary of the annotations for the cowpea SSH libraries that were extracted from SSHdb's sequence database. The data for the forward library is sorted by ER3 values, which represents the amount of up-regulation of the transcript in drought stressed IT96D-602 cowpea plants compared to the control treatment. Several genes with known roles in the stress response in other plants were present at high frequency in the cowpea SSH forward library with positive ER3 values, such as glutathione S-transferase (GST), a late embryogenesis abundant 5 protein (LEA), miraculin (MIR), thaumatin (THAU), pathogenesis related protein 1 (PR1), cowpea responsive to dehydration 2 (CPRD2), and a universal stress response protein (Additional file 5). Photosynthesis related genes had positive ER3 values in the reverse library screening indicating that their transcripts were up-regulated in the control treatment, which means they were down-regulated in the drought-stressed IT96D-602 cowpea plants (Additional file 5). Additional file 5 illustrates the usefulness of the output from SSHdb, showing the 14 redundant partner groups from the forward library and 26 redundant partner groups from the reverse library. For each group, the representative clone's ID is given, together with the number of redundant partners in that group. Also, each representative clone is labelled with its ER3 value, adjusted p-value, B-statistic and inverse ER2 value calculated by SSHscreen, as well as with a putative function corresponding to the Blast2GO annotations and priority selected BLAST result for each group added by SSHdb. The provision of BLASTN results (as well as BLASTX results) is very useful, since several of the priority annotations were BLASTN hits to rRNA of chloroplast or nuclear origin, indicating that some of the highly abundant non-coding RNA had been retained in the mRNA preparation and was cloned in the SSH library. This is most likely due to priming on A-rich tracts within non-coding RNA or self-priming of rRNA during cDNA synthesis [36].

Interestingly, inspection of the ER plots (Figure 2a and 2b) indicates that the majority of the genes (> 88%) that were cloned in both the forward and reverse SSH libraries have negative inverse ER2 values (present in quadrants I and IV). This indicates that most of the forward library clones were rare in the drought stressed IT96D-602 cowpea plants, and thus were enriched relative to other transcripts in this sample by the normalization step of the SSH process (Figure 2a). This is because a negative inverse ER2 value (log_2 _[UT/ST]) means that the amount of molecules of the gene is greater in ST (i.e. after subtraction) than in UT (before subtraction). The same is true for the reverse library clones, indicating the transcripts are rare relative to other transcripts in the control plants (Figure 2b).

Figure 4 shows the value of the ER plots to aid in the choice of non-redundant clones for sequencing. To illustrate this, we plotted the ER3 versus inverse ER2 values for a selection of clones from the eight largest redundant partner groups in the library (42 clones from the forward library and 26 from the reverse library). As indicated by the colour coding in Figure 4, clones from the same redundant partner groups clustered together. Drought stress up-regulated clones (ER3 > 0) encoding GST (mauve), THAU (red), PR1 (blue) and MIR (yellow) formed clusters that were relatively distinct, thus the choice of a few clones within each region is likely to capture the sequences for most genes in the library. Redundant partners of drought stress down-regulated clones (ER3 < 0 in Figure 4) also clustered together, namely lipid transfer protein (LTP; purple), LHCB4.3 light harvesting complex PSII (LHC; orange) and chlorophyll a/b-binding protein (CHL < 90 bp; green) (CHL > 170 bp; dark green). Clones encoding 26S rRNA (26S; blue) also clustered together with ER3 values close to 0. This indicates that 26S rRNA transcripts are present in similar quantities in the stressed and control cowpea plants, as expected, although non-coding RNA was not expected to be captured in either library.

### Verification of SSHscreen Enrichment Ratios using qPCR

**Figure 5:**
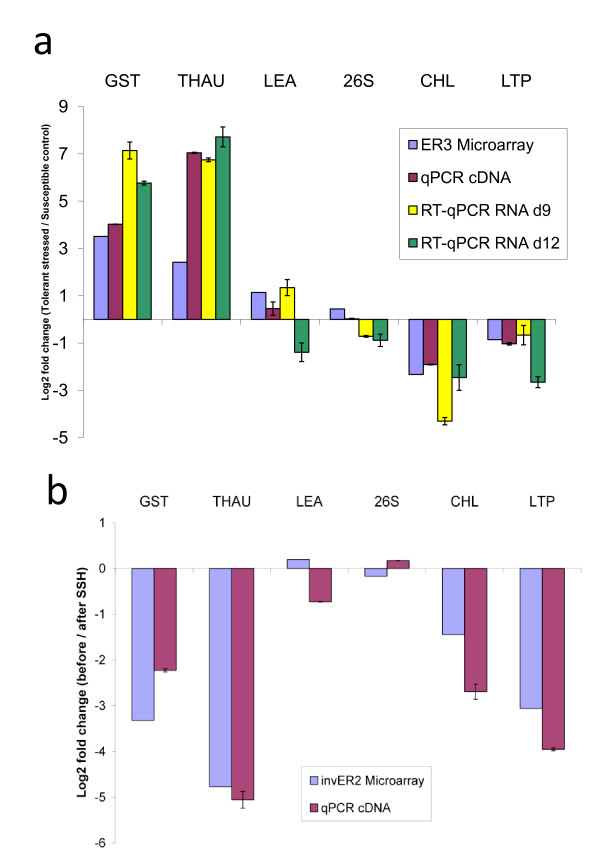
**Regulation (a) and relative abundance (b) of selected cowpea genes (qPCR verification)**. **(a) **Confirmation of differential expression in drought-stressed tolerant cowpea (IT96D-602) versus control susceptible cowpea (Tvu7778) observed in microarray studies. The expression ratios for each gene in the microarray experiment are indicated by blue bars and qPCR on cDNA by red bars. RT-qPCR using total RNA isolated from leaves after 9 and 12 days of stress treatment are indicated in yellow and green bars, respectively. **(b) **Confirmation that transcripts of selected genes had low abundance (i.e. rare) before subtraction. The log_2 _ratios before and after SSH are presented. Negative log_2 _ratios indicate that cDNAs have greater signals in subtracted samples compared to unsubtracted samples, indicating that they were rare before subtraction and have been enriched by the SSH process. Results from the microarray experiment are indicated by blue bars and the qPCR results by red bars. (Error bars = standard deviation of replicate qPCR experiments)

Representative SSH library clones of six cowpea genes were selected for verification of the SSHscreen enrichment ratios using qPCR. These were three up-regulated genes from the forward library (GST, THAU and LEA; Additional file 5), two down-regulated genes from the reverse library (CHL and LTP; Additional file 5), and 26S rRNA which was not differentially expressed (Additional file 5). qPCR corroborated the direction of gene regulation (ER3 value) calculated by SSHscreen analysis of the microarray data for all six selected genes (Figure 5a; compare blue to purple, yellow and green bars). Firstly, qPCR was carried out on the unsubtracted material used to construct the SSH libraries, the same material used to determine the ER3 values. The unsubtracted treated (UT) cDNA sample was a mixture of cDNA from drought stressed cowpea IT96D-602 at 9 and 12 days; and the unsubtracted control (UC) was a mixture of cDNA from control cowpea Tvu7778 at 9 and 12 days. After normalization of the qPCR data using the glyceraldehyde-3-phosphate dehydrogenase C-subunit (*gapC*) gene, an expression ratio was calculated (log_2_(drought stressed cowpea/control cowpea)). Good correlation between the ER3 values and the qPCR expression ratios was seen for all six genes (Figure 5a; compare blue bars with purple bars). GST, THAU and LEA were up-regulated, CHL and LTP were down-regulated and 26 S rRNA unchanged (Figure 5a). Secondly, since the libraries were constructed from mixtures of cDNA at two time points, reverse transcriptase quantitative PCR (RT-qPCR) was carried out on the RNA samples from the individual time points before they were pooled for SSH library construction (Figure 5a, yellow and green bars). GST and THAU were up-regulated, and CHL and LTP were down-regulated at both time points, thus corroborating the ER3 values (Figure 5a). Interestingly, LEA was up-regulated at 9 d and down-regulated at 12 d, and thus the transcript abundance measured in the mixtures used to make the SSH library is likely to be an average between the two (Figure 5a). RT-qPCR analysis of 26S rRNA at the two time points gave expression ratios that are essentially unchanged between the two treatments (Figure 5a).

The SSH process aims to equalize the proportion of genes in the final subtracted sample before cloning by enriching for rare transcripts and suppressing the amplification of highly abundant transcripts [37]. A rare gene before subtraction should be in increased amounts in the subtracted sample and *vice versa*. The inverse ER2 value [log_2_(UT/ST) for the forward library; log_2_(UC/SC) for the reverse library] provides a measure of this, since clones with inverse ER2 value < 0 are rare before subtraction (ie UT < ST or UC < SC). The cowpea drought expression forward and reverse libraries contained mostly rare clones, with inverse ER2 values < 0 (see Figures 2a and 2b; blue bars in Figure 5b). qPCR was also used to verify the SSHscreen inverse ER2 values using the same cDNA samples, and all five genes that were tested (GST, THAU, LEA, CHL and LTP) gave negative log_2_(before/after subtraction) values, and closely mirrored the inverse ER2 values, confirming that they were rare in the unsubtracted samples (compare purple with blue bars; Figure 5b). 26S rRNA transcripts are expected to be abundant in any plant cell, however the amount of rRNA in the cDNA sample derived from the mRNA isolation step is unlikely to be representative, since it is present due to false priming. Importantly, normalization of the qPCR data for verification of the ER2 values cannot be done with an endogenous housekeeping gene, since no product should be present in the same abundance before and after subtraction. Therefore, equal amounts of an alien gene fragment (human *beta-globin*) were spiked into the cDNA samples and effectively used for normalization of the qPCR data.

## Discussion

### SSHscreen-SSHdb pipeline facilitates the screening and annotation of SSH library clones

SSH remains a popular approach for gene discovery based on its advantages of enriching for genes that are differentially expressed between treatments, as well as the recovery of rare transcripts [10,38]. SSH has proven particularly useful as a first step in genomics research of non-model organisms that do not have genome sequence information [26,39]. In this study, we have developed two software tools, SSHscreen and SSHdb, which greatly facilitate gene discovery using SSH (Figure 1; Figure 3). Furthermore, we have demonstrated functionality of the SSHscreen-SSHdb pipeline with its application to the identification of drought-responsive genes from the non-model crop cowpea. Our approach represents a significant improvement compared to commonly used approaches in which SSH libraries are screened qualitatively using inverse dot blots, and sequence information is stored and managed on an individual researcher's desktop.

SSH libraries, constructed using either a commercial kit or homemade protocols, have the limitation that they often contain clones derived from transcripts that escaped subtraction (i.e. false positives), clones derived from highly abundant RNA species, such as rRNA, and some redundancy (i.e. the same inserts in several clones) [25]. For example, even though we performed the subtraction effectively, the forward and reverse SSH libraries constructed from cowpea plants in this study were calculated to have 9% and 46% false positives, respectively (negative ER3 values). Despite using mRNA for library construction, 6% of the clones in the reverse library were 26S rRNA, mostly likely due to self-priming or priming on A rich regions by the oligo-dT primer [36]. Approximately 67% of the sequenced clones were redundant. This means that sequencing all the clones from an SSH library would be a very inefficient use of resources, since many false positives and redundant clones would be sequenced. Commonly, this is overcome by first screening the SSH library clones as colonies or PCR products on nylon membranes using inverse dot blots [40]. This, however does not provide accurate quantification, and the choice of clones to use for normalization is difficult. This study provides an alternative approach of using a simple R package SSHscreen 2.0.1 to apply appropriate control spot normalization methods, and calculate differential expression ratios with statistical support after screening the SSH clones on a small number of microarray slides.

SSHscreen has the advantage over a basic limma analysis in that it is tailored for the screening of SSH libraries as a semi-automated process with appropriate background correction, normalization, differential expression analysis and false discovery rate corrections. In a single analysis, the user can submit microarray data for slides containing both forward and reverse libraries, hybridized with cDNA targets for both ER3 and ER2 calculations, and obtain top tables (i) of differentially expressed genes between the unsubtracted treatment and control, and (ii) with information on the relative abundance of cloned transcripts in these unsubtracted samples. Another output of SSHscreen is the ER3 vs inverse ER2 plot for each library (eg. Fig. 2), a feature not provided in a basic limma analysis.

### SSHscreen 2.0.1 improvements compared to SSHscreen 1.0.1

Several improvements have been made to SSHscreen 2.0.1 compared to the previous version [28]. Version 2.0.1 has a change to the terminology of cDNA samples, which improves clarity and is applicable to all biological contrasts. In SSHscreen 1.0.1, samples before subtraction were termed UT (unsubtracted tester) and UD (unsubtracted driver) [28]. However, this was confusing since UT referred to treated cDNA for a forward library and to control cDNA for a reverse library, and likewise for UD. In SSHscreen 2.0.1 the terms unsubtracted treated (UT), and unsubtracted control (UC) are used to provide a single term for each cDNA. In SSHscreen 1.0.1, the subtracted material used to construct the libraries were termed ST_F _and ST_R_, and these have been changed in SSHscreen 2.0.1 to subtracted treated (ST) and subtracted control (SC), respectively. This clarifies that the forward (ST) and reverse (SC) library are enriched for up-regulated and down-regulated transcripts, respectively, from the treatment under study.

Our previous study inferred a measure of the up-regulation in response to the treatment of each clone in the SSH library (UT/UD ratio) based on data calculated from two sets of microarray slides, as follows: log_2_(UT/UD) = [Enrichment ratio 1 (ER1)(log_2_(ST/UD)] - [(ER2)(log_2_(ST/UT)] [25]. This was implemented in SSHscreen 1.0.1 [28]. The current SSHscreen 2.0.1 approach supersedes the ER1 calculation, since ER3 (log_2_(UT/UC)) for forward library clones provides a direct measure of up-regulation between treated (UT) and control (UC) on a single set of microarray slides. Down-regulation of clones in the reverse library can also be calculated from the same microarray slide data (ie ER3 for reverse library clones = (log_2_(UC/UT)). SSHscreen 2.0.1 is able to accommodate analysis of both forward and reverse libraries spotted on the same slide (using the command library = "both").

Within-slide normalization of two-colour microarray data is an important consideration to account for systematic bias due to differences between the Cy3 and Cy5 dyes [30]. Commonly, loess normalization is applied [30], however this is based on the assumption that most of the genes on the array are not differentially expressed. This is legitimate for most whole genome microarray experiments, however it is not appropriate when the array is constructed from an SSH library, which selects for differentially expressed genes. SSHscreen 1.0.1 provided loess normalization as the default method, and SSHscreen 2.0.1 provides the improvement of spike-in control spot-based normalization with the option of giving full or partial weight to control spots when fitting the loess curve. Fardin et al [31] state that there are two main considerations for the spikes in this type of normalization of custom arrays. Firstly, the intensities of the spikes should span the range of intensities of the experimental data, and secondly, the strength of the loess curve with respect to the number of replicates. We applied full weight to the spike-in control spots in our normalization approach, and effective normalization can be seen in the boxplots of the control spots across the 12 slides (Additional file 3; similar to Figure 1 of [31]) which shows the variability of M values in the raw data is considerably diminished by the normalization. The spike-in control spots spanned 90-99% of the range of data intensities (see A values on the MA plots in Additional file 4), which is sufficient compared to the 75% range reported by [31]. Reliability of the normalization and strength of the loess curve through the control spots is further illustrated by our calculations that the average standard deviations of the M values for the spike-in controls across the 12 slides decreased from 0.18 to 0.10 after normalization, a similar improvement to that reported in Figure 2 of [31].

The outputs of SSHscreen are toptables of ER3 and inverse ER2 values which the user can rank based on moderated t-statistics and associated adjusted p-values calculated with limma functions [29]. Clones can be chosen for sequencing based on a positive ER3 value and a user-defined threshold of p-value adjusted for multiple testing [41]. In SSHscreen 2.0.1, the user has flexibility to use the B-statistic as an alternative for choosing clones. This is because the user can make a prior estimate of the percentage of differentially expressed genes, which is set at 1% default in limma for whole genome arrays and 50% default in SSHscreen 2.0.1. Changing this estimate does not change the moderated t-statistic rank, but changes the value of the B-statistic [32,33]. Clones with a postive B-statistic have more than a 50% chance of differential expression.

Enrichment ratio 2 is calculated in SSHscreen 2.0.1 as an inverse ER2 value (log_2_(UT/ST) for ease of interpretation in the ER3 versus inverse ER2 plots, since it arranges rare → abundant transcripts from left → right on the plot (Figure 2). It gives a measure of whether a clone in the library represents a transcript that was rare or abundant in the original tester sample, based on the theory of the SSH process that normalizes the relative amount of transcripts in the final subtracted tester sample that is cloned [10]. SSHscreen 2.0.1 provides a plot of the ER3 versus inverse ER2 values, which provides another tool in the selection of clones for sequencing. As shown in the current cowpea study (Figure 4), redundant clones clustered on the ER3 versus inverse ER2 plot, thus these plots can be used to choose clones for sequencing that are spatially separated. Interestingly, this plot was able to distinguish between longer and shorter clones of CHL (Figure 4). It should be noted that clusters do overlap (Figure 4), so although this plot serves to improve the efficiency of selecting unique clones, some redundant clones will be chosen.

### Microarray-derived SSHscreen enrichment ratios were confirmed by qPCR

We validated the ER3 and ER2 calculations derived from microarray hybridization signals using an independent technique, qPCR. Three cowpea genes, encoding GST, THAU, and LEA were significantly up-regulated more than 2-fold in the drought-stressed cowpea plants compared to the control plants (ER3 value > 1; adjusted p value < 0.05). qPCR of the UT and UC cDNA mixes prior to subtraction, as well as RT-qPCR of RNA from the individual time points used to make the UT and UC mixes confirmed the up-regulation of these three genes (Figure 5a). Interestingly, RT-qPCR showed that LEA was up-regulated at 9 d after initiation of drought stress and down-regulated at 12 d after drought stress, and thus the microarray and qPCR of the UT/UC mixes represent the average (Figure 5a). Similarly, ER3 values for two selected down-regulated genes, CHL and LTP were confirmed by qPCR and RT-qPCR. The 26S rRNA escaped subtraction in the construction of the reverse subtraction library and thus is observed to be at equal quantities in the UT and UC samples prior to subtraction (i.e. ER3 ~0) and this was also confirmed by the qPCR results (Figure 5a). The inverse ER2 values for all five selected differentially expressed genes were negative, indicating that their transcripts were rare in the original tester samples and had been enriched during the normalization step of the SSH process. qPCR confirmed this, indicating that the microarray hybridizations accurately reflect the relative amount of gene fragments in the target cDNA mixes (Figure 5b). Additional validations were done with 352 features on the microarray slides, and ER2 and ER3 data values correlated well with qPCR data (Pearson's correlation coefficients of 0.78 and 0.87, respectively; data not shown).

### SSHdb links SSHscreen data to sequence annotations for clones

The output from SSHscreen is a priority list of clones to sequence, and thus the next step is efficient management of the sequence information in the context of the SSHscreen results. Another tool SSHSuite was developed previously to manage sequence information from SSH libraries [16], however each user is required to install the software as well as the complete NCBI sequence database on a Linux workstation. Since it also lacked several functionalities, such as linkage to SSHscreen top table information, grouping of redundant clones, and customized export of data, we chose to develop SSHdb as a web-based tool with no software requirements for the user except an internet browser. Another advantage of our approach is that the complete NCBI sequence database is mirrored at a single site and, therefore, can be updated centrally.

SSHdb proved very effective in managing the sequence information for a set of sequences obtained from the forward and reverse drought-stressed cowpea libraries. Each clone was automatically annotated using two approaches, Blast2GO [34] and BLAST similarity searches of sequenced clones against the NCBI non-redundant nucleotide and peptide databases (nt/nr) [35]. Several features of SSHdb make it particularly effective for non-model organisms for which there is not an annotated genome sequence available. Blast2GO was designed as an annotation tool for non-model organisms and uses a more robust approach than BLAST to assign putative functional annotations to sequences as well as significant GO terms [34]. Providing BLASTN, as well as BLASTX hits, allows the identification of clones derived from non-coding RNA, which escaped the subtraction. This is a common problem in SSH library construction, as seen in our study with 6% clones derived from 26S rRNA. SSHdb also allows for manual curation of the annotations. The top ten BLAST hits sorted by E value are stored in the database, and the user is given the choice of choosing the representative annotation. Very often with non-model organisms the top hit is to a sequence that is not functionally annotated (e.g. "hypothetical protein", "expressed sequence"), whereas the second hit is to an annotated sequence, which can then provide the user with a working hypothesis of the putative identity of the clone. This was our experience for some of the cowpea clones in this study. In other studies, due to the poorly annotated rice genome in GenBank, we found the same problem with SSH clones from non-model monocots, pearl millet and banana, that had top hits to unannotated rice genes, whereas more useful hits within the top 10 were to sequences from other plants with annotations [26,39].

The SSHscreen data for each clone can be inspected in the SSHscreen toptable view, and annotated toptables or GAL files can be exported from SSHdb. This is particularly useful in cases where the same array is to be used later for gene expression profiling in a more in-depth study, for example over a time course of drought stress. Such an experiment could be analysed for differentially expressed genes using limma in R, for example, which would benefit from an annotated GAL file so that it could immediately be seen if differentially expressed clones had been sequenced. In this study, another feature of SSHdb was used to export the representative sequences of each redundant partner group in FASTA format with the correct header information, so that they could be submitted easily to dbEST at GenBank.

SSHdb is not limited to the management and analysis of sequences from SSH libraries, since it can organise any sequence dataset in FASTA format, including cDNA sequences from next generation sequencing projects. The cDNA Annotation System (CAS) is another generic tool for analysis of cDNA sequences [15], however it requires the complete NCBI database to be loaded and up-dated on individual desktops, and thus is less user-friendly for collaborative projects such as ours in which the co-workers are at different institutions.

### Identification of cowpea drought response genes

Confirming the importance of the cowpea genes identified in this study as role players in the drought response is beyond the scope of this paper, which focuses on a comprehensive description of the SSHscreen-SSHdb pipeline. However some inferences can be made by comparison with studies of stress responses in other plants. A glutathione S-transferase, a late embryogenesis abundant protein 5, and a universal stress response protein have clear links to drought stress responses. Glutathione S-transferase (EC 2.5.1.18; GST, group 1, Additional file 5) is an enzyme that catalyses the conjugation of reduced glutathione, via its sulfhydryl group, to the electrophilic centers on various substrates [42]. Glutathione is a tripeptide present in the intracellular space of plants and other organisms, functioning to keep sulfhydril groups reduced and to remove toxic metabolites. The induction of GST during drought stress in cowpea may protect the plant cells from a build-up of toxic compounds, thus contributing to its drought tolerance.

Late embryogenesis abundant (LEA, group 5, Additional file 5) proteins were initially discovered in desiccating plant seeds but have subsequently been described in various plants and plant tissues. They are associated with abiotic stress tolerance in plants, namely desiccation, salt and cold stress [43]. Their structure changes during dehydration from an unordered conformation, lacking in tertiary structure, to a folded structure which may protect the cell from collapse, stabilising membranes or protecting other proteins by acting as chaperones during periods of water stress. Most LEA proteins fall into three main groups, but two unnumbered groups were discovered in cotton: Lea5 and Lea14 [44]. These two are the only cloned cotton mRNAs encoding LEA's that are highly induced in drought-stressed leaves. They are predicted to be more hydrophobic and possibly more structured than LEA groups 1 - 3 [43]. LEA from the cowpea drought expression library in this study has the characteristic Lea5 motif (Pfam family PF03242 http://www.sanger.ac.uk/Software/Pfam), and is most similar to the drought-induced cotton Lea5 and a Lea5 protein identified in desiccating seeds of soybean (GenBank AAB38782).

The cowpea drought stressed forward library also contained a clone that matched a universal stress protein from *Arabidopsis thaliana *(TAIR: AT5G54430.1). These plant proteins have sequence similarity to UspA that has been well characterized in bacteria. Bacterial UspA is a small serine and threonine phosphoprotein that is induced by several stress treatments, and strains with mutations in this gene are less stress tolerant [45]. This may represent an ancient conserved stress mechanism at the cellular level. Iuchi *et al*. [46] identified genes induced after 5 h of dehydration in detached leaves of cowpea line IT84S-2246-4, and named them "cowpea clones responsive to dehydration" (CPRD). One of these genes (CPRD2) was also isolated in our study (Additional file 5).

Several pathogenesis-related genes were induced during drought stress in cowpea, namely a THAU, PR1, and a wound induced protein (WIN2). Overlap in the responses to biotic and abiotic stresses has been documented [47]. This may reflect a structural stabilizing role that these proteins may confer to protect against water loss and cellular damage by either stress. THAU, for example has the unique property of being a very sweet protein with a distinct protein structure made up of beta-sheets with a high content of beta-turns and very few alpha-helices.

The reverse library was dominated by clones encoding components of photosynthesis, such as chlorophyll a/b binding proteins (groups 15, 17 and 22, Additional file 5) [48,49], ribulose-1,5-bisphosphate carboxylase small subunit rbcS1, and the chloroplast genes fructose-bisphosphate aldolase 1 and phytoene synthase (Additional file 5). This reflects a reduction in photosynthesis during drought stress. Similar genes of the photosynthetic apparatus were also down-regulated in leaves of *P. vulgaris *under progressive drought stress [50]. They include carbonic anhydrase and the photosynthesis-related genes encoding ribulose 1,5-bisphosphate carboxylase (large and small subunits), chlorophyll a/b-binding protein CP24 precursor and photosystem I light-harvesting chlorophyll a/b-binding protein. Chlorophyll a/b-binding proteins are part of the light-harvesting complex that act as antennae to capture light excitation energy and deliver it to photosystems I and II. In *Arabidopsis*, *cab *genes were also more than 5-fold repressed under drought stress [51].

Three different lipid transfer proteins (LTP; group 20, 21 & 25, Additional file 5) were cloned in the reverse library. Plant LTPs show a highly conserved secondary structure, forming a hydrophobic pocket capable of carrying a fatty acid, phospholipid or acyl-CoA, and have been shown *in vitro *to transfer lipids between membranes [52]. Drought responsive LTPs have been described in *Solanum pennellii *[53]. Down-regulation of LTP during drought stress possibly indicates a need to suppress LTP mediated signalling.

The SSHscreen-SSHdb pipeline could be improved in future by developing a GUI version of SSHscreen, taking the user through a step-by-step analysis of the microarray data, similar to the limmaGUI version of limma [54]. Additionally, an integrated web-based package incorporating SSHscreen and SSHdb functionality could be developed, similar to WebArray [55].

## Conclusion

Although there are several alternative approaches such as cDNA-AFLP, DD-RT-PCR and RNA-Seq, SSH remains a popular approach for gene discovery from non-model organisms for which an annotated genome sequence is not available. It is particularly useful for laboratories focused on a particular research question without access to resources to conduct whole transcriptome sequencing using next generation technologies. We have developed the software SSHscreen 2.0.1 which facilitates the quantitative screening of clones in an SSH library from any biological system, and provides the user with a range of statistics to make effective choices of which clones to sequence. The sequence information is then stored and annotated in a web-accessible database, SSHdb, which project collaborators can readily access and interpret for future gene function studies. SSHscreen can be downloaded from http://microarray.up.ac.za/SSHscreen/. SSHdb is available at http://sshdb.bi.up.ac.za/.

## Methods

### Plant materials and treatments

Cowpea (*V. unguiculata *L. Walp) breeding lines IT96D-602 and Tvu7778 were provided by the Dr BB Singh of the International Institute of Tropical Agriculture (IITA) [19]. Seeds were germinated and plants were grown in a glasshouse under 11 h day length, 28°C and 18°C day and night temperatures, respectively, and watering three times weekly. At six weeks, five replicate plants of each variety were divided into two groups. One group was subjected to drought stress by withholding water, and the other group was kept to the control watering scheme.

### RNA extraction

RNA was isolated from cowpea leaves using Tri-reagent (Sigma) and Polyvinyl pyrrolidone (PVP) (Ambion's Plant RNA isolation aid). Contaminating genomic DNA was removed with the Turbo DNA-free kit (Ambion) and the RNA cleaned up with the Plant RNeasy kit (Qiagen, Hilden, Germany).

### Construction of cDNA library using SSH

Differential expression analysis by means of SSH [10] was employed to prepare a cDNA drought expression library for cowpea. Messenger RNA (mRNA) was isolated from 50 μg pools of stressed IT96D-602 RNA (9 and 12 days without water) (treated) and unstressed Tvu7778 RNA (9 and 12 days) (control) using an Oligotex mRNA purification kit (Qiagen). cDNA was synthesised from each mRNA using the cDNA synthesis system (Roche Diagnostics, Basel, Switzerland) to be used as unsubtracted testers and unsubtracted drivers in SSH [25]. Subtractive hybridisation was performed on *Rsa*I (Roche Diagnostics) -digested tester and driver cDNA fragments using the PCR-Select cDNA subtraction kit (BD Biosciences Clontech, Palo Alto, CA), as previously described [25]. A forward subtraction was performed by using the treated sample as tester (and control as driver), and a separate reverse subtraction was performed by using the control cDNA as tester (and treated cDNA as driver). After subtraction the products were amplified by a primary PCR and a nested secondary suppression PCR to generate differentially expressed cDNA fragments (termed ST (subtracted treated) and SC (subtracted control) for the forward and reverse libraries, respectively). Replicate PCR reactions were pooled, size fractionated and cloned into the pGEM-T Easy cloning vector and transformed into *Escherichia coli *JM109 following the manufacturers' instructions (Promega, Madison, WI). Transformed colonies were selected by blue-white selection on 100 μg/ml ampicillin LB-agar selection media (spread with X-Gal and IPTG) and stored as 25% glycerol stocks at -70°C in sterile 96-well culture plates (Corning, NY). In addition, unsubtracted PCR products from the treated cDNA (drought stressed IT96D-602) (termed UT) and control cDNA (control Tvu7778) (termed UC) were also prepared to be used for SSHscreen analysis as described in [28].

### Fabrication of SSH library on glass slide array

Inserts of the cowpea drought expression cDNA library were amplified with PCR directly from overnight bacterial cultures in 96-well format (Thermo-Fast, ABGene, Epsom, UK) in 100 μl reactions with 1 U Biotaq DNA polymerase (Bioline) and the SP6 and T7 primers (Additional file 6). The PCR plate was sealed with a silicon mat (Corning). Reactions were incubated in a PTC-100 Thermocycler (MJ Research) at 94°C for 5 min; 30 cycles of (94°C for 30 s, 50°C for 30 s and 72°C for 1 min); and 72°C for 5 min.

The PCR products were purified with Montage PCR purification plates on a vacuum manifold (Microsep) and resuspended in 50 μl SDW. The suspensions were transferred to 96-well storage plates, covered with well caps (Nunc, Roskilde, Denmark) and stored at -20°C. The purified PCR products were dried down in a vacuum centrifuge at 45°C, resuspended in 50% dimethyl sulfoxide (DMSO), transferred to 384-well spotting plates and stored at -70°C until microarray spotting.

The control genes *gfp *(717 bp fragment in pGEM-T Easy, positions 1603-2319 of GenBank accession number AF078810), *globin *(human *beta-globin*; 474 bp fragment in pBluescriptSK, positions 50-523 of NM_000518) and *nptII *(812 bp fragment in pGEM-T Easy, positions 142-953 of V00618) were purchased from the Nottingham Arabidopsis stock centre [NASC, http://arabidopsis.org.uk]. They were transformed into *E. coli *JM109 (Promega). An *its *clone in pGEM-T Easy (193 bp fragment from the internal transcribed spacer 2 of the rRNA genes from *Leptographium elegans*) was also used as a control gene. It matches to positions 268-458 of AF343675.1. Plasmids were isolated from cultures using the Qiaspin miniprep plasmid isolation kit (Qiagen). PCR products of the four control genes were prepared using the T7 and SP6 primers (PCR product sizes: *gfp *(893 bp), *nptII *(988 bp), *its *(369 bp)) or the T7 and M13R primers for *globin *(677 bp). Montage purified PCR products of twelve 100 μl PCR reactions each were pooled, concentrated and transferred to 12 wells each of a 384-well spotting plate. An equal volume of DMSO was added so that the final concentrations in 50% DMSO ranged from 70-100 ng/μl. Five two-fold serial dilutions were also prepared for each PCR fragment (*gfp*: 180-11.25 ng/μl;*globin*: 100-6.25 ng/μl;*nptII*: 150-9.375 ng/μl; and *its*: 130-8.125 ng/μl), transferred to an additional 10 wells per fragment, an equal volume of DMSO added, and spotted on the glass slides.

Glass slides (Corning GAPS II) were spotted with the cowpea drought expression library (4160 clones in total from the forward and reverse libraries) and controls using the Array Spotter Generation III (Molecular Dynamics, Sunnyvale, CA) at the University of Pretoria, Pretoria, South Africa http://microarray.up.ac.za. Each sample spot was duplicated on the slide in replicate blocks on either side of the slide, and therefore replicates are not spatially close together. The slides were allowed to dry overnight in the protective atmosphere of the spotter, after which the DNA was cross-linked under ultraviolet (UV) light for 3 min. The slides were stored in a desiccator covered in foil at room temperature.

### Screening SSH library on microarrays

SSH cDNA fragments (ST, SC, UT and UC), purified by PCR Minelute cleanup kit (Qiagen), were digested with *RsaI *(10 U per microgram DNA) in the appropriate buffer overnight at 37°C. The fragments were separated from the adaptor fragments by electrophoresis on a 1.5% low melting point agarose gel (Seaplaque, FMC Bioproducts) in 0.5× TAE and purified from the gel using the Qiaquick gel extraction kit (Qiagen).

The control fragments were excised from their plasmids using restriction digestion to exclude any T7 and SP6 primer binding sites (*Kpn*I/*Xba*I for *globin *(product of 548 bp); *Nco*I/*Pst*I for *gfp *(768 bp); *Eco*RI for *nptII *(830 bp) and *its *(211 bp)). Restriction fragments were purified with the Qiaquick gel extraction kit (Qiagen). Each target sample of SSH cDNA fragments (200 ng) were spiked with equal amounts of a control fragment pool made up of different quantities of four control fragments (45 ng *globin*, 45 ng *its*, 4.5 ng *nptII *and 0.45 ng *gfp*) for within-slide normalization. Spiking with equal amounts of fifteen- or three-fold dilutions of the control fragment pool were tested and also gave sufficient hybridization for within-slide normalization (data not shown).

Targets were labelled by direct Cy-dUTP incorporation by Klenow enzyme (Fermentas, Vilnius, Lithuania). Each SSH fragment sample was labelled with both dyes (Cy3 and Cy5) for a dye-swap experiment of each slide. The protocols and data analysis techniques described in [28] were followed, with some modifications. DNA to be labelled, in a volume of 12 μl, was denatured at 95°C for 5 min and placed on ice. The following were added to the pairs of denatured DNA samples to yield a total reaction volume of 20 μl: 2 μl of 10× Klenow buffer (Fermentas); 2 μl 10× Hexanucleotide mix (Roche Diagnostics); 2 μl Klenow enzyme (5 U/μl; Fermentas); 2 μl of a dNTP mix containing 1 nmol each of dATP, dCTP and dGTP, 0.74 nmol dTTP and 0.27 nmol of either Cy3-dUTP or Cy5-dUTP (Amersham Biosciences). The labelling reaction was incubated overnight (17-20 h) at 37°C. The labelled DNA was cleaned up from unincorporated dye using the Qiaquick PCR purification kit (Qiagen). Dye incorporation was measured using a NanoDrop ND-1000 UV-Vis Spectrophotometer (Nanodrop Technologies, Wilmington, DE).

Labelled SSH targets were combined in pairs using equal amounts of Cy3 and Cy5 dye incorporation for each target in each pair required for SSHscreen analysis. Each labelled target DNA mix was dried down in a vacuum centrifuge at 45°C and resuspended in 50 μl hybridisation solution (50% formamide, 25% 4× Microarray hybridisation buffer (Amersham Biosciences), 25% SDW). Labelled targets in hybridisation solution were denatured at 95°C for 2 min and placed on ice.

Glass slides arrayed with the SSH cDNA libraries were pre-treated in 1% bovine serum albumin (BSA; Roche Diagnostics), 3.5× SSC (525 mM sodium chloride and 52.5 mM sodium citrate) and 0.2% sodium dodecyl sulphate (SDS) at 60°C for 20 min. After rinsing in SDW at room temperature, the slide was dried by centrifugation in a 50 ml tube at 1000 × g for 4 min at room temperature in a swing-out rotor (Eppendorf 5810R centrifuge). The slide was placed in a locally manufactured hybridisation chamber (HybUP, NB Engineering, Pretoria, South Africa) with 20 μl SDW in the reservoirs on either side. Labelled and denatured target was applied to the slide and gently overlaid with a cover slip. The chamber was sealed and incubated in a water bath at 42°C for 16 h. Slides were washed for 4 min at 42°C with 1× SSC (150 mM NaCl, 15 mM sodium citrate)/0.2% SDS, twice with 0.1× SSC (15 mM NaCl, 1.5 mM sodium citrate)/0.2% SDS and three washes of 0.1× SSC for 1 min at room temperature. After dipping the slide in SDW at room temperature and centrifuged to dry, it was immediately scanned using a GenePix™ 4000B scanner (Axon Instruments, Foster City, CA).

GenePix Pro 5.1 software (Axon Instruments) was used to automatically locate all the spot positions from the scanner-generated TIFF images and associate them with each specific clone in a GenePix Array List (GAL file)(available at NCBI GEO Accession # GSE20273). The GAL file links the information from the arraying process to the analysis, since it provides identification information for each spot printed on the slide. Bad quality spots (irregularly shaped or with hybridisation artefacts; signal/noise ratio < 3) were flagged for exclusion during data analysis and the array of circles were manually adjusted for a better fit. GenePix Pro 5.1 was used to extract the dye intensity data of each spot and save the data for each slide in a GenePix Results file (gpr).

### SSHscreen software analysis of microarray data

The SSHscreen 2.0.1 package, written as a single function in the R programming language, was used for analyzing the resulting microarray data to calculate ER3 and inverse ER2 values. ER3 values (log_2_(UT/UC) for the forward library clones; and log_2_(UC/UT) for the reverse library clones) quantify differential expression of transcripts that give rise to the clones in each library [28]. Inverse ER2 values (log_2_(UT/ST) for the forward library clones; and log_2_(UC/SC) for the reverse library clones) reflect the relative abundance of transcripts for each clone in the unsubtracted samples [28]. The original version of SSHscreen is described in [28]. Improvements to the functionality were added to the original R code, the documentation was updated and the latest version was packaged as SSHscreen 2.0.1. SSHscreen can be downloaded at http://microarray.up.ac.za/SSHscreen/, together with a demo data set and an example R script. For details on how to use SSHscreen and to view a full description of all the possible argument options, type *help(SSHscreen) *at the R command line (after loading the SSHscreen library). The SSHscreen analysis implemented by the R script provided as Additional file 7 is described in the following sections:

### SSHscreen pre-processing

SSHscreen analysis in R (version 2.8.1) required the libraries of limma (version 2.16.5) and SSHscreen 2.0.1 to be installed in R. The input data to SSHscreen 2.0.1 were the 12 Genepix results files from hybridization experiments to the cowpea SSH library arrays (*gpr *files deposited at NCBI GEO series accession number GSE20273), with the Targets file, Spot types file (Additional files 8 & 9, respectively) and the GAL file (available at GEO accession number GSE20273).

The first step was to weight all spots that had been flagged as poor quality (signal/noise < 3) by the GenePix Pro 5.1 image analysis programme http://www.axon.com so that these spots would not be used to calculate the normalization factors. Background correction used the normexp method in limma [56] with an offset of 50, which dampens the variation of the log-ratios for very low intensity spots towards zero. This approach is encouraged specifically when using empirical Bayes methods from the limma package [33].

Within array normalization was based on data from the four alien controls (*globin*, *its, nptII *and *gfp*) that had been spotted as dilution series on each array to make up a total of 176 control spots/array. Equal amounts of each control fragment had been added to pairs of target samples to be labelled with the Cy3 and Cy5 dyes, and thus hybridization signals from the control spots could be used for within-slide normalization. We used this data to apply the up-weighting print-tip loess within-array normalization method of limma in SSHscreen 2.0.1., which essentially applies full weight to all the control spots and zero weight to the spots of the probes from the SSH library. Thereby a loess curve is fitted through the control spots, which normalizes the data within each slide to remove the systematic errors due to the dye effects [30]. Between-slide normalization was carried out using the Aquantile method, which is based on the assumption that the distribution of A values (eg. Average expression: 1/2(log_2 _[UT*UC]) is similar across all arrays. MA plots (Additional file 4) can be exported at each stage of pre-processing.

### SSHscreen enrichment ratio analysis and outputs

ER3 and inverse ER2 values for each SSH library clone were calculated in SSHscreen using the functions of limma for differential gene expression analysis [33]. ER3 values were based on testing the null hypothesis that there is no differential expression for a gene between the UT and UC samples, whereas inverse ER2 values test the null hypothesis that there is no difference in abundance of a clone between the unsubstracted sample (e.g. UT) and its corresponding subtracted sample (e.g. ST). This was achieved in SSHscreen 2.0.1 by implementing the limma function that fits gene-wise linear models through the normalized expression data that was the output of pre-processing. Thereafter, an empirical Bayes approach is used in limma to calculate a moderated t-statistic for each gene, in which the standard errors have been moderated across all the genes on the array. This approach of variance shrinkage improves inference about each gene in experiments in which there are a low number of replicates [33]. We adjusted for multiple testing using Benjamini & Hochberg's method [41] for controlling the false discovery rate, which computes an adjusted p-value for the hypothesis test of each gene. A prior guess of 50% differentially expressed genes for the SSH libraries was implemented as the default in SSHscreen 2.0.1, and was used in calculating B-statistics for each gene using limma functions. The B-statistic [32,33] can be interpreted as the log-odds that a specific gene is differentially expressed. Thus a positive B-statistic represents more than a 50-50 chance of differential expression. The outputs of SSHscreen were top tables which reported the enrichment ratios for each gene and associated statistics, namely the moderated t-statistic, associated p-value adjusted for multiple testing, and B-statistic (Additional file 5), MA-plots (Additional file 4) and a graphical representation of each clone on ER-plots (Figures 2a and 2b), which were used to select clones for sequencing.

### Sequencing

Selected cowpea drought expression library clones were sequenced using the T7 Promoter primer by Inqaba Biotec (SA) or Macrogen (USA). Colonies were sent on LB-agar plates containing 100 μg/ml ampicillin. Non-redundant sequences have been deposited in dbEST at Genbank (Accession numbers GR942571 - GR942610).

### Annotation and management of sequences using SSHdb

SSHdb (available at http://sshdb.bi.up.ac.za) was developed as a web-based tool for sequence management of clones in SSH libraries. The SSHdb interface was written using Turbogears [57], a Python web application framework. Currently, a central MySQL database is used to store sequence, top table and annotation information. SQLAlchemy [58], an object relational mapper for Python and toolkit for SQL, is implemented within SSHdb when a user queries the database.

For each input sequence in FASTA format, SSHdb removed the vector and adaptor fragments after BLASTN [35] searches were performed against the NCBI UniVec database http://www.ncbi.nlm.nih.gov/VecScreen/UniVec.html. Further BLASTN searches were carried out against all sequences already uploaded in the database, so that redundant partners in the library (using a BLASTN E-value cut-off value of 10e-10) could be identified. For each redundant partner group, the longest sequence in the group was selected by default as the representative clone. Multiple sequence alignments (generated by ClustalW [59]) for individual redundant partner groups could be viewed and downloaded from SSHdb. For each representative clone, SSHdb performed nucleotide-nucleotide and translated sequence comparisons using BLASTN and BLASTX searches against a local installation of the NCBI non-redundant nucleotide and peptide databases (nt/nr) [35]. For cases where the E-value of the top BLASTX hit was less than 10e-10), this hit was automatically selected as the default priority annotation. The top 10 BLASTX and BLASTN hits were stored in the database. Blast2GO [34] has also been implemented in SSHdb, and the putative GO annotations for representative sequences are recorded in SSHdb. SSHdb provides two major views of the data, the SSH database view, which shows the annotated representative clones (see Additional file 1), or the SSH toptable view, which shows the enrichment ratio data for each clone in each library (see Table 1). SSHdb can be updated as additional clones are sequenced.

### Quantitative PCR

qPCR primer pairs (20-mers) were designed from selected cowpea sequences to amplify products between 120 and 250 bp in length from the SSH cDNA fragment pools (UT, UC, ST, and SC) (Additional file 6). qPCR reactions containing 1× Sensimix (Quantace, Celtic Molecular Diagnostics), SYBR Green, 2.5 mM MgCl_2_, the appropriate primer pair (200 nM each) and cDNA template in a total volume of 25 μl were set up and run on a Rotor-Gene (Corbett Research). The enzyme was activated by a hold at 95°C for 10 min followed by 45 cycles of 95°C for 15 s, annealing at 56°C for 30 s and extension at 72°C for 6 s. SYBR green fluorescence was measured after the extension step of every cycle. qPCR was performed on serial ten-fold dilutions of a mix of UT and UC cDNAs (templates ranging from 0.5 pg to 50 ng) to construct standard curves for each primer pair. The quantification cycle (Cq) values from the qPCR fluorescent profiles were converted to input nanograms of template using the standard curves. Average nanogram quantities for each gene was normalised relative to the data for the respective sample's reference gene content.

For ER3 verification, qPCR was performed in duplicate on 50 ng each of cDNA from the two cowpea cultivars before subtraction (UT and UC). Glyceraldehyde-3-phosphate dehydrogenase C-subunit (*gapC*) was used as a reference gene. A consensus sequence between the *gapC *genes of *Medicago truncatula *[GenBank: AC135505_Mt, exons only], *G. max *[GenBank: DQ192668_Gmax1 and DQ355800_Gmax2] and *Pisum sativum *[GenBank: PEAGAPCI] was used to design the *gapC *reference gene primers (Additional file 6). An expression ratio of log_2_(ng in UT/ng in UC) was calculated for each gene.

For ER2 verification, normalization of qPCR results between unsubtracted and subtracted cDNA samples (i.e. UT and ST; UC and SC) required the spiking of cDNA samples with equal amounts of an alien gene. qPCR was performed in duplicate on 10 ng of the UT, ST; UC and SC cDNA templates, each spiked with 50 pg of the human *beta-globin *fragment (prepared using the Globin forward and reverse primers, Additional file 6). Average input nanogram quantities were calculated for each gene and normalised with the *globin *spike content. Expression ratios of log_2_(ng in UT/ng in ST) and log_2_(ng in UC/ng in SC) were calculated for each gene.

RT-qPCR reactions were performed in duplicate using 100 ng of RNA isolated prior to SSH library construction from drought-treated IT96D-602 cultivar and control Tvu7778 at two time points, 9 and 12 days, separately. The Sensimix One-step RT-qPCR kit (Quantace) was used with a reverse transcription step at 49°C for 30 min inserted before the cycling profile described above. The quantification cycle (Cq) values from the RT-qPCR fluorescent profiles were converted to input nanograms of template using the standard curves.

## Competing interests

The authors declare that they have no competing interests.

## Authors' contributions

NC developed the SSHscreen and SSHdb software, analysed the microarray and sequence data and drafted the manuscript. IG constructed the SSH library, performed the microarray hybridizations and qPCR experiments, participated in the microarray and sequence data analysis and drafted the manuscript. DO contributed to conceiving the study, the design of the biological experiments and assisted in writing the manuscript. DKB was instrumental in the design of the study, development of the software, interpretation of data, and drafted the manuscript. All authors have read and approved the final manuscript.

## Supplementary Material

Additional file 1 **Screenshot of the SSHdb 'Sequence Database' view**. The SSHdb screenshot shows a summary of some of the redundant partner groups of the sequenced clones in the cowpea SSH library http://sshdb.bi.up.ac.za/. For each group, the representative clone ID, the priority BLAST annotation and the number of redundant partners in the group are given, as well as a tick box allowing individual groups to be marked so that corresponding sequence and/or annotation information can be exported. By clicking on the representative clone ID, the user can view and select the preferred annotation from the top 10 BLASTX or BLASTN hits, download the multiple sequence alignment of the clones in that group and change the representative clone if required. Click here for file

Additional file 2 **Example of Microarray pseudocolour images following hybridization**. Example of a cowpea microarray image following hybridization with differentially labelled cDNA samples, and scanning with a GenePix™ 4000B scanner (Axon Instruments). In this particular example, subtracted treated (ST) (cDNA prepared from pooled RNA extracted from IT96D-602 cowpea plants drought stressed for 9 and 12 days, and subtracted with cDNA prepared from RNA isolated from control Tvu7778 plants) was labelled with Cyanine™-3 dye, (green pseudocolour). Unsubtracted treated cDNA (prepared from pooled RNA extracted from IT96D-602 cowpea plants drought stressed for 9 and 12 days) was labelled with Cyanine™-5 dye (red pseudocolour). **(b) **Dye swap of the experiment in Additional file 2a. Subtracted treated (ST) cDNA was labelled with Cyanine™-5 dye, and unsubtracted treated cDNA was labelled with Cyanine™-3 dye. These differentially labelled cDNA samples were hybridised to the cowpea microarray slide and scanned with a GenePix™ 4000B scanner (Axon Instruments). Click here for file

Additional file 3 **Box plots of M-values of control spots before and after normalization for all 12 slides used for SSHscreen analysis**. Each box corresponds to one array. **(a) **Box plots before normalization (i.e. only background subtracted M-values of raw data). **(b) **Box plots after within and between slide normalization (i.e. spike-in control spot loess and A-quantile normalized M-values). Click here for file

Additional file 4 **MA plots after normalization of the forward and reverse cowpea SSH libraries**. M versus A plots for microarray slides after within and between slide normalization in SSHscreen 2.0.1. For each comparison of interest, there were four technical replicates of which two were dye-swaps: plots a-d (forward library, UT versus UC), e-h (forward library, UT versus ST), i-l (reverse library, UC versus UT), and m-p (reverse library, UC versus SC). Forward and reverse library clones are indicated by blue and yellow dots, respectively. Control spots are indicated as red, light blue, green or mauve dots. M and A values were calculated as described in [24], for example (a) M = log_2_(Cy5 labelled sample = UT)/(Cy3 labelled sample = UC); A = (log_2_(UT*UC))/2; and for example (e) M = log_2_(Cy5 labelled sample = UT)/(Cy3 labelled sample = ST); A = (log_2_(UT* ST))/2. Click here for file

Additional file 5 **Cowpea drought responsive genes annotated in SSHdb (after sequencing of selected clones)**. Table of sequenced cowpea drought responsive genes from the forward and reverse libraries with annotations in SSHdb derived from Blast2GO and BLAST analysis, as well as SSHscreen enrichment ratio values. Data is shown for representative clones from each redundant partner group. Click here for file

Additional file 6 Table of oligonucleotide primers used in this study Click here for file

Additional file 7 **SSHscreen R script used for ER3 analysis of cowpea SSH libraries**. R script used for ER3 analysis of both forward and reverse cowpea SSH libraries together after loading the limma 2.16.5 and SSHscreen 2.0.1 libraries in R 2.8.1. The working directory contained the Targets file (Additional file 8), SpotTypes file (Additional file 9), the 12 Genepix Results (gpr) and the GenePix Array List (GAL) file (available at GEO accession number GSE20273). Click here for file

Additional file 8 **Targets file used for ER3 analysis of cowpea SSH libraries**. Targets file listing the raw microarray slide data used for the SSHscreen ER3 analysis described in Additional file 7. Click here for file

Additional file 9 **SpotTypes file used for ER3 analysis of cowpea SSH libraries**. SpotTypes file defining the control and cDNA spot types used for the SSHscreen ER3 analysis described in Additional file 7. Click here for file
