# Supplementary figures and images for: SSHscreen and SSHdb, generic software for microarray based gene discovery: application to the stress response in cowpea
Source: Plant Methods. 2010 Apr 1;6:10. doi: 10.1186/1746-4811-6-10 (PMC2859861; doi:10.1186/1746-4811-6-10)

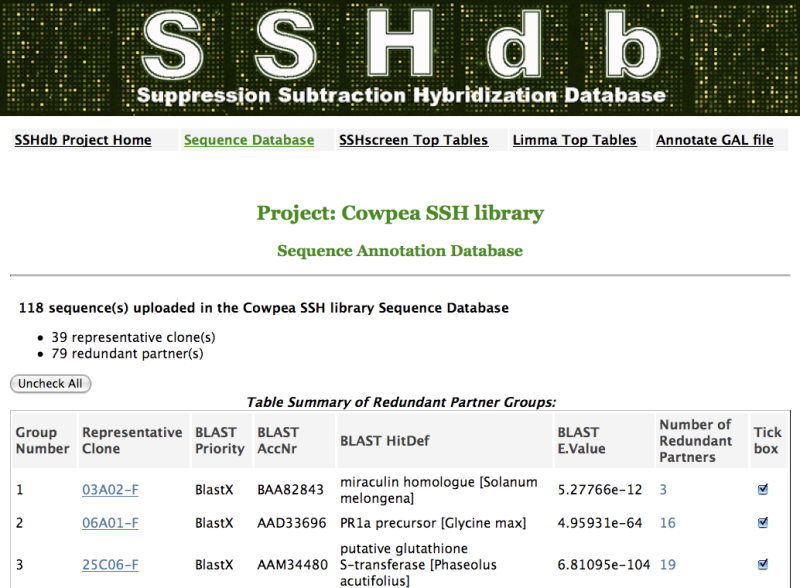

Supplement: Additional file 1 — Screenshot of the SSHdb 'Sequence Database' view. The SSHdb screenshot shows a summary of some of the redundant partner groups of the sequenced clones in the cowpea SSH library http://sshdb.bi.up.ac.za/. For each group, the representative clone ID, the priority BLAST annotation and the number of redundant partners in the group are given, as well as a tick box allowing individual groups to be marked so that corresponding sequence and/or annotation information can be exported. By clicking on the representative clone ID, the user can view and select the preferred annotation from the top 10 BLASTX or BLASTN hits, download the multiple sequence alignment of the clones in that group and change the representative clone if required. [file 1746-4811-6-10-S1.TIFF]

a

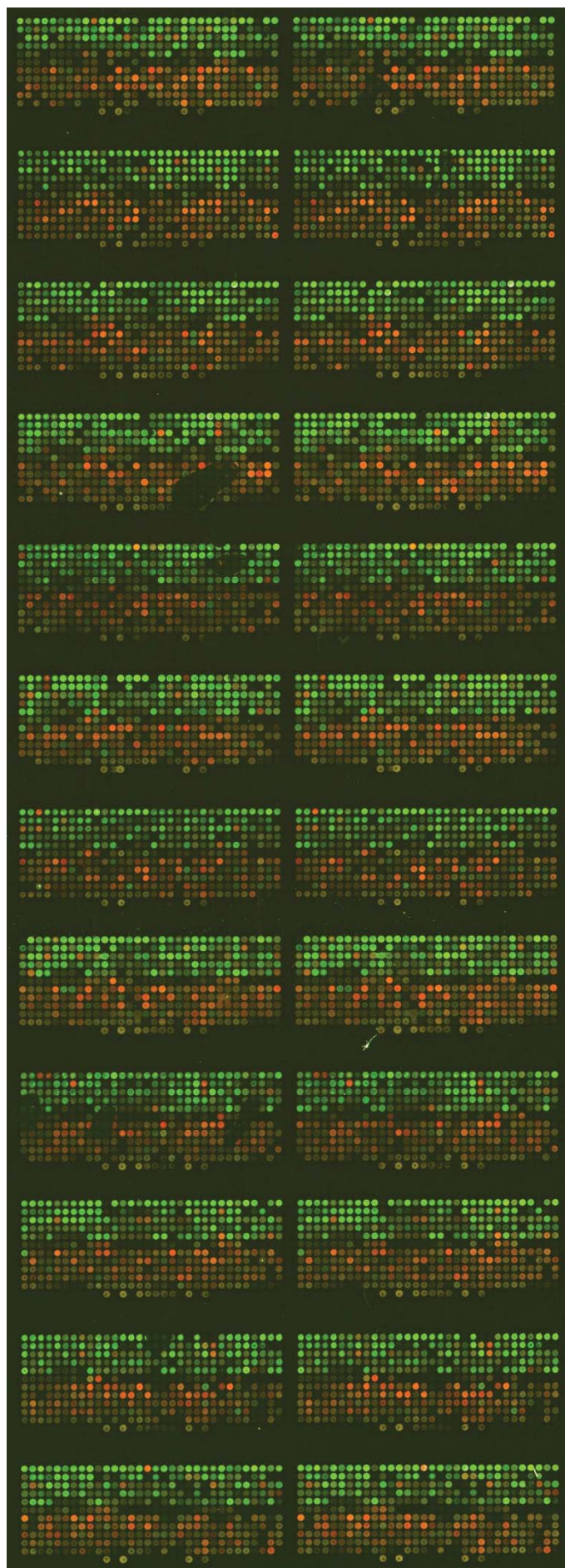

b

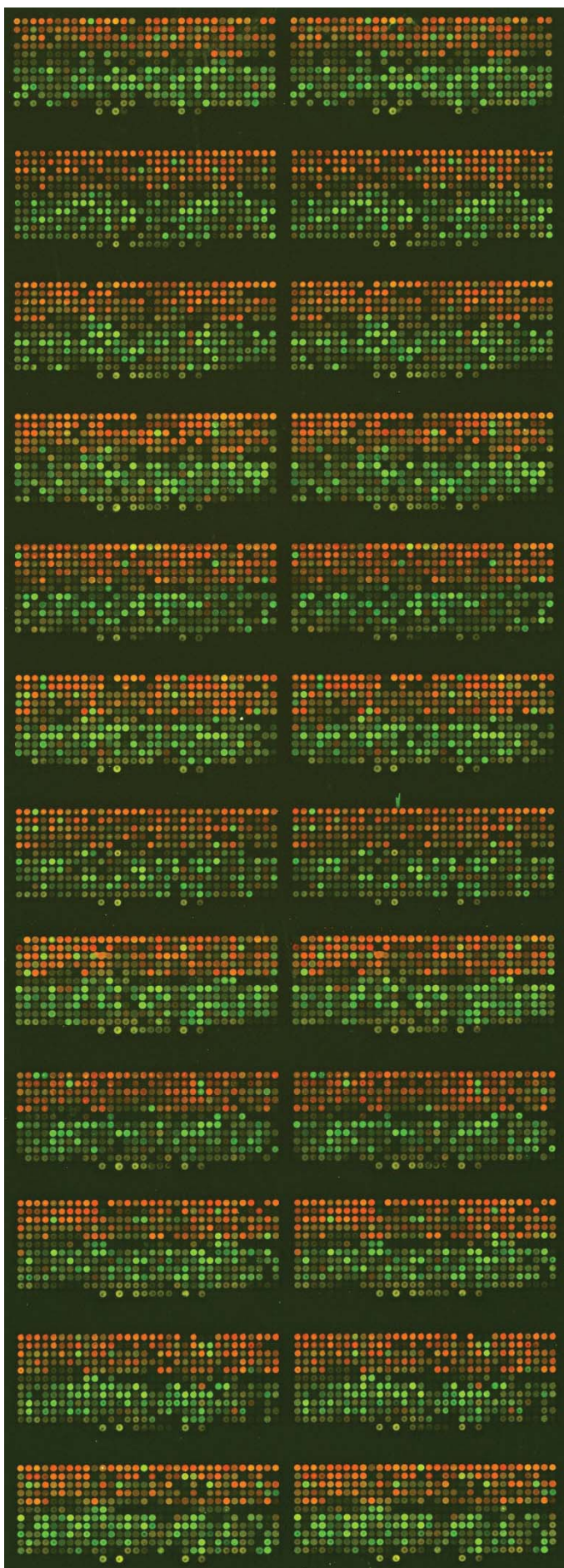

Supplement: Additional file 2 — Example of Microarray pseudocolour images following hybridization. Example of a cowpea microarray image following hybridization with differentially labelled cDNA samples, and scanning with a GenePix™ 4000B scanner (Axon Instruments). In this particular example, subtracted treated (ST) (cDNA prepared from pooled RNA extracted from IT96D-602 cowpea plants drought stressed for 9 and 12 days, and subtracted with cDNA prepared from RNA isolated from control Tvu7778 plants) was labelled with Cyanine™-3 dye, (green pseudocolour). Unsubtracted treated cDNA (prepared from pooled RNA extracted from IT96D-602 cowpea plants drought stressed for 9 and 12 days) was labelled with Cyanine™-5 dye (red pseudocolour). (b) Dye swap of the experiment in Additional file 2a. Subtracted treated (ST) cDNA was labelled with Cyanine™-5 dye, and unsubtracted treated cDNA was labelled with Cyanine™-3 dye. These differentially labelled cDNA samples were hybridised to the cowpea microarray slide and scanned with a GenePix™ 4000B scanner (Axon Instruments). [file 1746-4811-6-10-S2.PDF]

(a)

### Before normalization: control spots

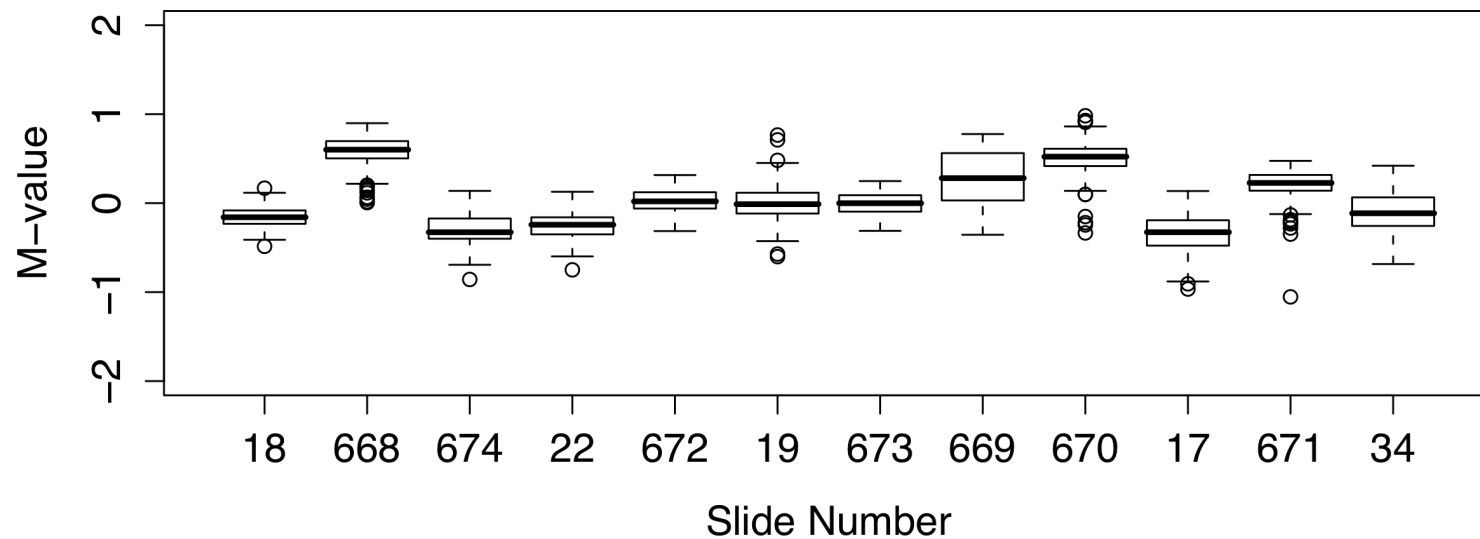

(b)

### After normalization: control spots

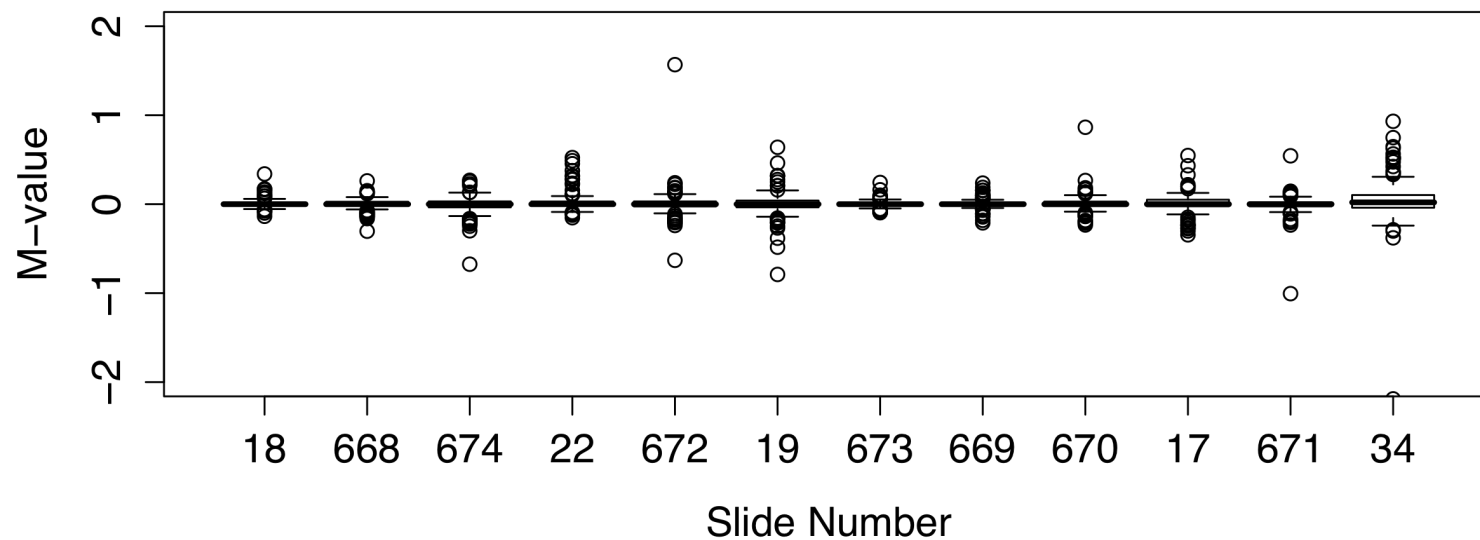

Supplement: Additional file 3 — Box plots of M-values of control spots before and after normalization for all 12 slides used for SSHscreen analysis. Each box corresponds to one array. (a) Box plots before normalization (i.e. only background subtracted M-values of raw data). (b) Box plots after within and between slide normalization (i.e. spike-in control spot loess and A-quantile normalized M-values). [file 1746-4811-6-10-S3.PDF]
