# Supplementary material for: SSHscreen and SSHdb, generic software for microarray based gene discovery: application to the stress response in cowpea
Source: Plant Methods. 2010 Apr 1;6:10. doi: 10.1186/1746-4811-6-10 (PMC2859861; doi:10.1186/1746-4811-6-10)
Supplement: Additional file 4 — MA plots after normalization of the forward and reverse cowpea SSH libraries. M versus A plots for microarray slides after within and between slide normalization in SSHscreen 2.0.1. For each comparison of interest, there were four technical replicates of which two were dye-swaps: plots a-d (forward library, UT versus UC), e-h (forward library, UT versus ST), i-l (reverse library, UC versus UT), and m-p (reverse library, UC versus SC). Forward and reverse library clones are indicated by blue and yellow dots, respectively. Control spots are indicated as red, light blue, green or mauve dots. M and A values were calculated as described in [24], for example (a) M = log2(Cy5 labelled sample = UT)/(Cy3 labelled sample = UC); A = (log2(UT*UC))/2; and for example (e) M = log2(Cy5 labelled sample = UT)/(Cy3 labelled sample = ST); A = (log2(UT* ST))/2. [file 1746-4811-6-10-S4.PDF]

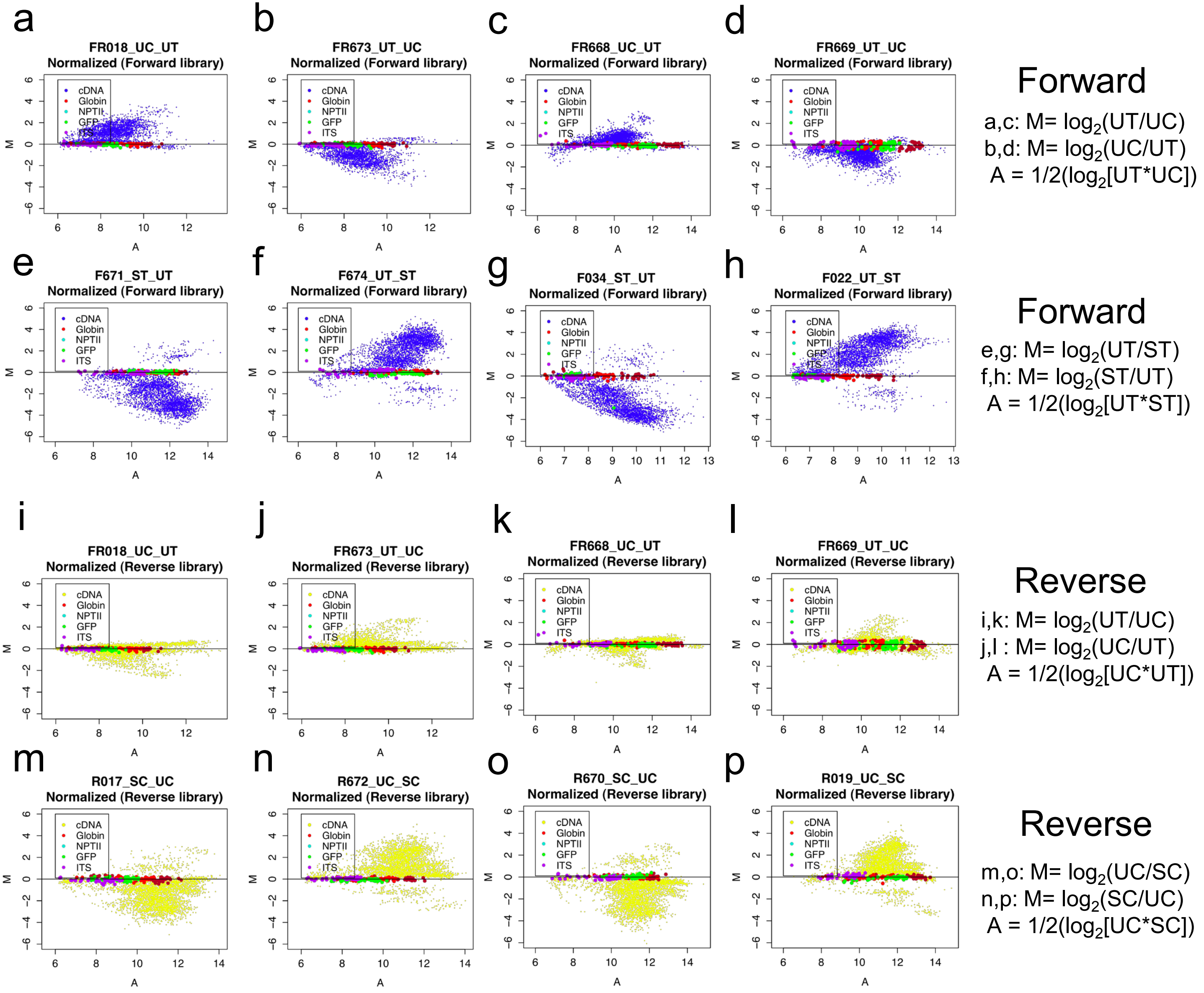

a
FR018_UC_UT
Normalized (Forward library)
b
FR673_UT_UC
Normalized (Forward library)
c
FR668_UC_UT
Normalized (Forward library)
d
FR669_UT_UC
Normalized (Forward library)
Forward
a,c: $M = \log_2(UT/UC)$
b,d: $M = \log_2(UC/UT)$
$A = 1/2(\log_2[UT*UC])$
e
F671_ST_UT
Normalized (Forward library)
f
F674_UT_ST
Normalized (Forward library)
g
F034_ST_UT
Normalized (Forward library)
h
F022_UT_ST
Normalized (Forward library)
Forward
e,g: $M = \log_2(UT/ST)$
f,h: $M = \log_2(ST/UT)$
$A = 1/2(\log_2[UT*ST])$
i
FR018_UC_UT
Normalized (Reverse library)
j
FR673_UT_UC
Normalized (Reverse library)
k
FR668_UC_UT
Normalized (Reverse library)
l
FR669_UT_UC
Normalized (Reverse library)
Reverse
i,k: $M = \log_2(UT/UC)$
j,l : $M = \log_2(UC/UT)$
$A = 1/2(\log_2[UC*UT])$
m
R017_SC_UC
Normalized (Reverse library)
n
R672_UC_SC
Normalized (Reverse library)
o
R670_SC_UC
Normalized (Reverse library)
p
R019_UC_SC
Normalized (Reverse library)
Reverse
m,o: $M = \log_2(UC/SC)$
n,p: $M = \log_2(SC/UC)$
$A = 1/2(\log_2[UC*SC])$
cDNA
Globin
NPTII
GFP
ITS
M
A
