# Supplementary material for: SSHscreen and SSHdb, generic software for microarray based gene discovery: application to the stress response in cowpea
Source: Plant Methods. 2010 Apr 1;6:10. doi: 10.1186/1746-4811-6-10 (PMC2859861; doi:10.1186/1746-4811-6-10)
Supplement: Additional file 5 — Cowpea drought responsive genes annotated in SSHdb (after sequencing of selected clones). Table of sequenced cowpea drought responsive genes from the forward and reverse libraries with annotations in SSHdb derived from Blast2GO and BLAST analysis, as well as SSHscreen enrichment ratio values. Data is shown for representative clones from each redundant partner group. [file 1746-4811-6-10-S5.PDF]

Additional file 5 - Cowpea drought responsive genes annotated in SSHdb (after sequencing of selected clones)

Forward library

BLAST2GO annotations				SSHscreen annotations				SSHdb annotations						
Group number	Representative clone ID	Sequence description	GO-terms	logFC (ER3)*	adj.P.Val	B	invER2	Vector free sequence (bp)	BLAST priority (X/N)	BLAST AccNr	BLAST Hit Def	BLAST Eval	RedPartner
1	25C06-F	glutathione s-transferase	P: defense response to bacterium F: glutathione transferase activity C: vacuole F: copper ion binding C: plasma membrane C: chloroplast stroma C: apoplast F: glutathione binding	2.92	4.E-08	17.5	-2.92	705	X	AAM34480	glutathione S-transferase [*Phaseolus acutifolius*] [GST]	7.E-104	19
2	46D11-F	protein	C: extracellular region	2.10	1.E-05	10.0	-4.04	543	X	AAD33696	PR1a precursor [*Glycine max*] [PR1]	5.E-64	16
3	33D04-F	thaumatin-like protein	C: extracellular region P: defense response	2.08	5.E-05	8.1	-4.91	780	X	DQ269446	thaumatin-like [*G. max*] [THAU]	2.E-66	5
4	26H07-F	wound-induced protein win2	P: defense response to bacterium F: chitinase activity P: chitin catabolic process F: protein binding P: defense response to fungus F: chitin binding P: cell wall macromolecule catabolic process P: response to salt stress	1.69	1.E-06	14.0	-2.9	524	X	P09762	wound-induced protein 2 [*Solanum tuberosum*]	3.E-63	2
5	07F09-F	late embryogenesis abundant protein	P: response to stress	1.15	5.E-07	14.9	0.05	473	X	AAB38782	late embryogenesis abundant 5 [*G.max*] [LEA]	1.E-27	0
6	46E07-F	19-like protein	C: cytoplasmic membrane-bounded vesicle	1.10	2.E-04	6.4	-0.81	538	X	AAU14999	MtN19-like protein [*Pisum sativum*]	7.E-73	0
7	29F12-F	kunitz-type protease inhibitor kpi-f4	F: endopeptidase inhibitor activity	1.06	1.E-04	7.0	-3.47	404	X	BAA82840	miraculin [*Youngia japonica*] [MIR]	2.E-10	3
8	26B01-F	protein	F: FAD binding F: oxidoreductase activity	0.82	7.E-04	5.1	-2.92	360	X	BAB33033	CPRD2 [*Vigna unguiculata*]	2.E-60	0
9	26B07-F	ammonium transporter	C: membrane P: ammonium transport	0.50	9.E-04	4.9	-3.45	516	X	NP_193087	ammonium transmembrane transporter [*Arabidopsis thaliana*]	1.E-63	0
10	29D02-F	universal stress protein family	C: cytosol P: response to stress C: plastid	0.30	3.E-02	1.1	-1.96	359	X	NP_563888	universal stress protein (USP) [*A.thaliana*]	1.E-14	0
11	30B02-F	---	---	0.25	3.E-02	0.9	-2.64	487	X	NP_564894	zinc finger (MYND type) family protein / F-box [*A.thaliana*]	9.E-10	0
12	14B10-F	calmodulin-binding protein	F: calmodulin binding	0.22	1.E-01	-0.5	-1.75	276	X	AAN65367	calmodulin-binding protein 60-A [*Phaseolus vulgaris*]	3.E-11	0
13	46G04-F	protein	C: cytoplasmic membrane-bounded vesicle F: protein binding C: membrane	0.17	1.E-01	-0.7	-1.12	161	N	AK285943	cDNA, clone: GMFL01-19-A06 [*G. max*]	1.E-48	0
14	31A10-F	---	---	-0.09	3.E-01	-1.4	1.84	356	N	AF479110	26S ribosomal RNA [*Medicago sativa*]	5.E-176	0

*ER3 for forward library calculated as log_2(drought stressed/control)

Reverse library

		BLAST2GO annotations		SSHscreen annotations				SSHdb annotations					
Group number	Representative clone ID	Sequence description	GO-terms	logFC (ER3)*	adj.P.Val	B	invER2	Vector free sequence (bp)	BLAST priority (X/N)	BLAST AccNr	BLAST Hit Def	BLAST Eval	RedPartner
15	16C12-R	light-harvesting complex ii protein lhcb1	P: photosynthesis, light harvesting C: photosystem II F: magnesium ion binding P: protein-chromophore linkage F: chlorophyll binding C: integral to membrane C: chloroplast C: photosystem I	1.99	5.E-06	11.6	-1.13	653	X	AAA50172	photosystem II chlorophyll a/b-binding (type I) [*G.max*] [CHL]	3.E-120	10
16	16B08-R	plastidic aldolase	P: glycolysis F: fructose-bisphosphate aldolase activity C: chloroplast C: mitochondrion	1.53	2.E-05	10.47	-1.24	570	X	Q01516	Fructose-bisphosphate aldolase 1,chloroplastic [*P.sativum*]	4.E-92	2
17	38A07-R	chlorophyll a b binding protein	C: membrane C: thylakoid F: protein binding P: photosynthesis, light harvesting C: chloroplast	1.32	2.E-05	10.29	-2.35	432	X	NP_181539	LHCB4.3(light harvesting complex PSII)[*A.thaliana*] [LHC]	2.E-43	4
18	17C04-R	---	---	1.17	3.E-03	5.3	-2.75	439	X	ACA23202	Kunitz trypsin inhibitor p20-1 [*G. max*]	9.E-14	1
19	37F06-R	ribulose -bisphosphate carboxylase	P: reductive pentose-phosphate cycle F: ribulose-bisphosphate carboxylase activity F: monooxygenase activity P: photorespiration P: oxidation reduction C: chloroplast C: mitochondrion	0.94	4.E-04	7.73	-0.9	369	X	AAG24882	ribulose-1,5-bisphosphate carboxylase rbcS1 [*G.max*]	1.E-23	0
20	36G04-R	---	---	0.76	1.E-04	8.95	-2.94	669	X	AAQ74628	lipid transfer protein II [*Vigna radiata*] [LTP]	1.E-30	3
21	38A10-R	lipid transfer protein precursor	P: defense response to fungus P: lipid transport P: defense response to Gram-positive bacterium F: lipid binding F: transporter activity	0.75	5.E-03	4.39	-2.08	422	X	ABF06565	non-specific lipid transfer-like protein [*Prosopis juliflora*]	2.E-16	0
22	21F04-R	type iii chlorophyll a b-binding protein	P: photosynthesis, light harvesting C: photosystem II F: magnesium ion binding P: protein-chromophore linkage F: chlorophyll binding C: integral to membrane C: photosystem I C: chloroplast thylakoid membrane C: mitochondrion	0.46	2.E-02	2.44	-1.67	492	X	1609235A	chlorophyll a/b binding protein 8 [*Solanum lycopersicum*]	1.E-61	1
23	42G12-R	---	---	0.43	2.E-01	-0.05	-3.2	505			No significant similarity		0
24	16F09-R	---	---	0.30	3.E-03	5.15	-0.48	521	N	EU196765	23S chloroplast rRNA [*P. vulgaris*]	0.00E+00	1
25	35F12-R	lipid transfer protein	F: lipid binding P: lipid transport	0.29	1.E-01	0.49	-2.78	523	X	Q43019	lipid-transfer protein 3 [*Prunus dulcis*]	2.00E-31	0

26	15F11-R	---	---	0.19	2.E-01	0.09	2.99	620	N	AF479110	26S ribosomal RNA [*M. sativa*]	5.E-176	1
27	24B10-R	pg1 protein	C: chloroplast	0.18	3.E-01	-0.4	-2.82	569	N	EU196765	16S chloroplast rRNA [*P. vulgaris*]	0.E+00	5
28	36A11-R	carbonic anhydrase	F: zinc ion binding C: chloroplast stroma F: carbonate dehydratase activity P: carbon utilization	0.04	8.E-01	-1.5	-2.39	611	X	AAD27876	carbonic anhydrase [*V. radiata*]	1.E-100	0
29	43B08-R	---	---	-0.05	7.E-01	-1.4	-0.69	144			No significant similarity		0
30	36A10-R	---	---	-0.09	4.E-01	-1.1	-4.31	766	X	AAG13810	RNA-binding protein Virp1a [*S. lycopersicum*]	7.E-49	1
31	18C04-R	signal recognition particle 54 kda subunit	F: mRNA binding P: SRP-dependent cotranslational protein targeting to membrane, signal sequence recognition F: GTP binding F: protein binding C: signal recognition particle, endoplasmic reticulum targeting F: nucleoside-triphosphatase activity F: 7S RNA binding	-0.10	3.E-01	-0.5	-2.02	305	X	P49972	Signal recognition particle SRP54 [*S. lycopersicum*]	5.E-23	0
32	38E10-R	protein	F: protein binding	-0.10	3.E-01	-0.6	-2.53	254	X	NP_190638	leucine-rich repeat family protein [*A.thaliana*]	2.E-26	0
33	16B07-R	aldo keto reductase	P: oxidation reduction F: oxidoreductase activity C: plastid	-0.11	2.E-01	-0.1	-3.65	723	X	NP_565656	aldo/keto reductase family protein [*A.thaliana*]	1.E-109	0
34	21H08-R	---	---	-0.13	5.E-01	-1.1	-2.48	746	X	NP_568178	proline-rich family protein [*A.thaliana*]	2.E-23	0
35	43E12-R	prolyl endopeptidase	P: proteolysis F: serine-type endopeptidase activity	-0.13	3.E-01	-0.6	-0.28	147	X	AAL86330	prolyl endopeptidase [*A.thaliana*]	1.E-13	0
36	21D09-R	at1g06530-like protein	C: plastid	-0.14	1.E-01	0.3	-3.77	437	X	ABH03547	ubiquitin-interacting factor 7 [*A.thaliana*]	2.E-14	0
37	24G10-R	unknown [Glycine max]	P: biosynthetic process F: transferase activity	-0.16	1.E-01	0.6	-1.97	323	X	Q9SSU8	Phytoene synthase, chloroplastic [*Daucus carota*]	3.E-02	0
38	43G10-R	cell elongation protein	F: FAD binding F: oxidoreductase activity F: metallopeptidase activity F: zinc ion binding	-0.16	7.E-02	1.0	-0.36	115	X	AAK15493	brassinosteroid biosynthetic protein LKB [*P. sativum*]	3.E-13	0
39	37B02-R	---	---	-0.34	2.E-02	2.7	-0.82	541	N	EU196765	23S chloroplast rRNA [*P. vulgaris*]	0.E+00	0
40	38A04-R	---	---	-0.40	9.E-04	6.8	-0.16	695	N	AF479105	26S ribosomal RNA [*Juglans nigra*] [26S]	0.E+00	5

#ER3 for reverse library calculated as log_2(control/drought stressed)

Each redundant partner *Group* (column 1) is represented by a *Representative clone ID* (column 2). By default SSHdb selects the clone that is the longest sequence from the group. Columns 3-4 give the Blast2GO consensus annotation and GO terms. Columns 5-8 give the regulation (*ER3*), statistical support for the ER3 value (*adj.P.Val* and *B*) and abundance (*invER2*) for each group calculated by SSHscreen. Columns 9-11 are annotations added by SSHdb for each group: *Length* is the length of the representative clone sequence after vector and adaptor fragments are removed, *Priority* indicates whether the BLASTN or BLASTX hit was selected (this can be manually curated), *BLAST* Accession number *(AccNr),* Hit Definition *(HitDef)* and E-value (*Eval*) are for the selected BLAST hit (from the top 10 hits) from either BLASTX/BLASTN results, *RedPartners* are the number of redundant partner clones in that group (grouped by SSHdb after performing local BLAST searches with a E-value cut off of 1E^{-10})
