# Supplementary material for: SSHscreen and SSHdb, generic software for microarray based gene discovery: application to the stress response in cowpea
Source: Plant Methods. 2010 Apr 1;6:10. doi: 10.1186/1746-4811-6-10 (PMC2859861; doi:10.1186/1746-4811-6-10)
Supplement: Additional file 6 — Table of oligonucleotide primers used in this study [file 1746-4811-6-10-S6.PDF]

Additional file 6: Table of oligonucleotide primers used in this study

Primer code	Forward primer (5' – 3')	Reverse primer (5' – 3')	Source (library clone number or GenBank accession number)	Expected product length (bp)
SP6	ATTTAGGTGACACTATAG			
T7	TAATACGACTCACTATAGGG			
M13R	CAGGAAACAGCTATGACC			
GST	GCTGGTGAAGGTGTTGGATA	CCACGATGGTCTGCTACTTA	25B06-F	199
THAU	AAGGTTCAGTTGCGCCACAG	AATCCGTCCACGTTGCTCAC	33E07-F	147
LEA	CCGTCTCCTTCTTCCTCAGT	TGCACCATCTCTTGTCACAG	07F09-F	163
26S	GGAATCGAGAGCTCCAAGTG	GTTGATTCGGCAGGTGAGTT	38G04-R	198
CHL	CTCATCCACGCTCAGAGCAT	CTGGACGAAGAAGCCGAACA	44C07-R	240
LTP	GCATCAGCGGTATCAACCTC	CCTCCTTGCCATCTCTTCCT	36F07-R	147
Globin	GGAGAAGTCTGCCGTTACTG	GCCATGAGCCTTCACCTTAG	NM_000518	175
GAPC	ATCAGCCAAGGACTGGAGAG	ACGGAATGCCATACCAGTCA	Consensus from: AC135505_Mt (exons only), DQ192668, DQ355800, PEAGAPCI	130
